# Search for new physics in dijet angular distributions using proton–proton collisions at $\sqrt{s}=13\hbox {TeV}$ and constraints on dark matter and other models

**DOI:** 10.1140/epjc/s10052-018-6242-x

**Published:** 2018-09-28

**Authors:** A. M. Sirunyan, A. Tumasyan, W. Adam, F. Ambrogi, E. Asilar, T. Bergauer, J. Brandstetter, E. Brondolin, M. Dragicevic, J. Erö, A. Escalante Del Valle, M. Flechl, M. Friedl, R. Frühwirth, V. M. Ghete, J. Grossmann, J. Hrubec, M. Jeitler, A. König, N. Krammer, I. Krätschmer, D. Liko, T. Madlener, I. Mikulec, E. Pree, N. Rad, H. Rohringer, J. Schieck, R. Schöfbeck, M. Spanring, D. Spitzbart, A. Taurok, W. Waltenberger, J. Wittmann, C.-E. Wulz, M. Zarucki, V. Chekhovsky, V. Mossolov, J. Suarez Gonzalez, E. A. De Wolf, D. Di Croce, X. Janssen, J. Lauwers, M. Pieters, M. Van De Klundert, H. Van Haevermaet, P. Van Mechelen, N. Van Remortel, S. Abu Zeid, F. Blekman, J. D’Hondt, I. De Bruyn, J. De Clercq, K. Deroover, G. Flouris, D. Lontkovskyi, S. Lowette, I. Marchesini, S. Moortgat, L. Moreels, Q. Python, K. Skovpen, S. Tavernier, W. Van Doninck, P. Van Mulders, I. Van Parijs, D. Beghin, B. Bilin, H. Brun, B. Clerbaux, G. De Lentdecker, H. Delannoy, B. Dorney, G. Fasanella, L. Favart, R. Goldouzian, A. Grebenyuk, A. K. Kalsi, T. Lenzi, J. Luetic, T. Seva, E. Starling, C. Vander Velde, P. Vanlaer, D. Vannerom, R. Yonamine, T. Cornelis, D. Dobur, A. Fagot, M. Gul, I. Khvastunov, D. Poyraz, C. Roskas, D. Trocino, M. Tytgat, W. Verbeke, B. Vermassen, M. Vit, N. Zaganidis, H. Bakhshiansohi, O. Bondu, S. Brochet, G. Bruno, C. Caputo, A. Caudron, P. David, S. De Visscher, C. Delaere, M. Delcourt, B. Francois, A. Giammanco, G. Krintiras, V. Lemaitre, A. Magitteri, A. Mertens, M. Musich, K. Piotrzkowski, L. Quertenmont, A. Saggio, M. Vidal Marono, S. Wertz, J. Zobec, W. L. Aldá Júnior, F. L. Alves, G. A. Alves, L. Brito, G. Correia Silva, C. Hensel, A. Moraes, M. E. Pol, P. Rebello Teles, E. Belchior Batista Das Chagas, W. Carvalho, J. Chinellato, E. Coelho, E. M. Da Costa, G. G. Da Silveira, D. De Jesus Damiao, S. Fonseca De Souza, H. Malbouisson, M. Medina Jaime, M. Melo De Almeida, C. Mora Herrera, L. Mundim, H. Nogima, L. J. Sanchez Rosas, A. Santoro, A. Sznajder, M. Thiel, E. J. Tonelli Manganote, F. Torres Da Silva De Araujo, A. Vilela Pereira, S. Ahuja, C. A. Bernardes, L. Calligaris, T. R. Fernandez Perez Tomei, E. M. Gregores, P. G. Mercadante, S. F. Novaes, Sandra S. Padula, D. Romero Abad, J. C. Ruiz Vargas, A. Aleksandrov, R. Hadjiiska, P. Iaydjiev, A. Marinov, M. Misheva, M. Rodozov, M. Shopova, G. Sultanov, A. Dimitrov, L. Litov, B. Pavlov, P. Petkov, W. Fang, X. Gao, L. Yuan, M. Ahmad, J. G. Bian, G. M. Chen, H. S. Chen, M. Chen, Y. Chen, C. H. Jiang, D. Leggat, H. Liao, Z. Liu, F. Romeo, S. M. Shaheen, A. Spiezia, J. Tao, C. Wang, Z. Wang, E. Yazgan, H. Zhang, J. Zhao, Y. Ban, G. Chen, J. Li, Q. Li, S. Liu, Y. Mao, S. J. Qian, D. Wang, Z. Xu, Y. Wang, C. Avila, A. Cabrera, C. A. Carrillo Montoya, L. F. Chaparro Sierra, C. Florez, C. F. González Hernández, M. A. Segura Delgado, B. Courbon, N. Godinovic, D. Lelas, I. Puljak, P. M. Ribeiro Cipriano, T. Sculac, Z. Antunovic, M. Kovac, V. Brigljevic, D. Ferencek, K. Kadija, B. Mesic, A. Starodumov, T. Susa, M. W. Ather, A. Attikis, G. Mavromanolakis, J. Mousa, C. Nicolaou, F. Ptochos, P. A. Razis, H. Rykaczewski, M. Finger, M. Finger, E. Carrera Jarrin, Y. Assran, S. Elgammal, S. Khalil, S. Bhowmik, R. K. Dewanjee, M. Kadastik, L. Perrini, M. Raidal, C. Veelken, P. Eerola, H. Kirschenmann, J. Pekkanen, M. Voutilainen, J. Havukainen, J. K. Heikkilä, T. Järvinen, V. Karimäki, R. Kinnunen, T. Lampén, K. Lassila-Perini, S. Laurila, S. Lehti, T. Lindén, P. Luukka, T. Mäenpää, H. Siikonen, E. Tuominen, J. Tuominiemi, T. Tuuva, M. Besancon, F. Couderc, M. Dejardin, D. Denegri, J. L. Faure, F. Ferri, S. Ganjour, S. Ghosh, A. Givernaud, P. Gras, G. Hamel de Monchenault, P. Jarry, C. Leloup, E. Locci, M. Machet, J. Malcles, G. Negro, J. Rander, A. Rosowsky, M. Ö. Sahin, M. Titov, A. Abdulsalam, C. Amendola, I. Antropov, S. Baffioni, F. Beaudette, P. Busson, L. Cadamuro, C. Charlot, R. Granier de Cassagnac, M. Jo, I. Kucher, S. Lisniak, A. Lobanov, J. Martin Blanco, M. Nguyen, C. Ochando, G. Ortona, P. Paganini, P. Pigard, R. Salerno, J. B. Sauvan, Y. Sirois, A. G. Stahl Leiton, Y. Yilmaz, A. Zabi, A. Zghiche, J.-L. Agram, J. Andrea, D. Bloch, J.-M. Brom, E. C. Chabert, C. Collard, E. Conte, X. Coubez, F. Drouhin, J.-C. Fontaine, D. Gelé, U. Goerlach, M. Jansová, P. Juillot, A.-C. Le Bihan, N. Tonon, P. Van Hove, S. Gadrat, S. Beauceron, C. Bernet, G. Boudoul, N. Chanon, R. Chierici, D. Contardo, P. Depasse, H. El Mamouni, J. Fay, L. Finco, S. Gascon, M. Gouzevitch, G. Grenier, B. Ille, F. Lagarde, I. B. Laktineh, H. Lattaud, M. Lethuillier, L. Mirabito, A. L. Pequegnot, S. Perries, A. Popov, V. Sordini, M. Vander Donckt, S. Viret, S. Zhang, T. Toriashvili, Z. Tsamalaidze, C. Autermann, L. Feld, M. K. Kiesel, K. Klein, M. Lipinski, M. Preuten, M. P. Rauch, C. Schomakers, J. Schulz, M. Teroerde, B. Wittmer, V. Zhukov, A. Albert, D. Duchardt, M. Endres, M. Erdmann, S. Erdweg, T. Esch, R. Fischer, A. Güth, T. Hebbeker, C. Heidemann, K. Hoepfner, S. Knutzen, M. Merschmeyer, A. Meyer, P. Millet, S. Mukherjee, T. Pook, M. Radziej, H. Reithler, M. Rieger, F. Scheuch, D. Teyssier, S. Thüer, G. Flügge, B. Kargoll, T. Kress, A. Künsken, T. Müller, A. Nehrkorn, A. Nowack, C. Pistone, O. Pooth, A. Stahl, M. Aldaya Martin, T. Arndt, C. Asawatangtrakuldee, K. Beernaert, O. Behnke, U. Behrens, A. Bermúdez Martínez, A. A. Bin Anuar, K. Borras, V. Botta, A. Campbell, P. Connor, C. Contreras-Campana, F. Costanza, V. Danilov, A. De Wit, C. Diez Pardos, D. Domínguez Damiani, G. Eckerlin, D. Eckstein, T. Eichhorn, A. Elwood, E. Eren, E. Gallo, J. Garay Garcia, A. Geiser, J. M. Grados Luyando, A. Grohsjean, P. Gunnellini, M. Guthoff, A. Harb, J. Hauk, M. Hempel, H. Jung, M. Kasemann, J. Keaveney, C. Kleinwort, J. Knolle, I. Korol, D. Krücker, W. Lange, A. Lelek, T. Lenz, K. Lipka, W. Lohmann, R. Mankel, I.-A. Melzer-Pellmann, A. B. Meyer, M. Meyer, M. Missiroli, G. Mittag, J. Mnich, A. Mussgiller, D. Pitzl, A. Raspereza, M. Savitskyi, P. Saxena, R. Shevchenko, N. Stefaniuk, H. Tholen, G. P. Van Onsem, R. Walsh, Y. Wen, K. Wichmann, C. Wissing, O. Zenaiev, R. Aggleton, S. Bein, V. Blobel, M. Centis Vignali, T. Dreyer, E. Garutti, D. Gonzalez, J. Haller, A. Hinzmann, M. Hoffmann, A. Karavdina, G. Kasieczka, R. Klanner, R. Kogler, N. Kovalchuk, S. Kurz, V. Kutzner, J. Lange, D. Marconi, J. Multhaup, M. Niedziela, D. Nowatschin, T. Peiffer, A. Perieanu, A. Reimers, C. Scharf, P. Schleper, A. Schmidt, S. Schumann, J. Schwandt, J. Sonneveld, H. Stadie, G. Steinbrück, F. M. Stober, M. Stöver, D. Troendle, E. Usai, A. Vanhoefer, B. Vormwald, M. Akbiyik, C. Barth, M. Baselga, S. Baur, E. Butz, R. Caspart, T. Chwalek, F. Colombo, W. De Boer, A. Dierlamm, N. Faltermann, B. Freund, R. Friese, M. Giffels, M. A. Harrendorf, F. Hartmann, S. M. Heindl, U. Husemann, F. Kassel, S. Kudella, H. Mildner, M. U. Mozer, Th. Müller, M. Plagge, G. Quast, K. Rabbertz, M. Schröder, I. Shvetsov, G. Sieber, H. J. Simonis, R. Ulrich, S. Wayand, M. Weber, T. Weiler, S. Williamson, C. Wöhrmann, R. Wolf, G. Anagnostou, G. Daskalakis, T. Geralis, A. Kyriakis, D. Loukas, I. Topsis-Giotis, G. Karathanasis, S. Kesisoglou, A. Panagiotou, N. Saoulidou, E. Tziaferi, K. Kousouris, I. Papakrivopoulos, I. Evangelou, C. Foudas, P. Gianneios, P. Katsoulis, P. Kokkas, S. Mallios, N. Manthos, I. Papadopoulos, E. Paradas, J. Strologas, F. A. Triantis, D. Tsitsonis, M. Csanad, N. Filipovic, G. Pasztor, O. Surányi, G. I. Veres, G. Bencze, C. Hajdu, D. Horvath, Á. Hunyadi, F. Sikler, T. Á. Vámi, V. Veszpremi, G. Vesztergombi, N. Beni, S. Czellar, J. Karancsi, A. Makovec, J. Molnar, Z. Szillasi, M. Bartók, P. Raics, Z. L. Trocsanyi, B. Ujvari, S. Choudhury, J. R. Komaragiri, S. Bahinipati, P. Mal, K. Mandal, A. Nayak, D. K. Sahoo, S. K. Swain, S. Bansal, S. B. Beri, V. Bhatnagar, S. Chauhan, R. Chawla, N. Dhingra, R. Gupta, A. Kaur, M. Kaur, S. Kaur, R. Kumar, P. Kumari, M. Lohan, A. Mehta, S. Sharma, J. B. Singh, G. Walia, A. Bhardwaj, B. C. Choudhary, R. B. Garg, S. Keshri, A. Kumar, Ashok Kumar, S. Malhotra, M. Naimuddin, K. Ranjan, Aashaq Shah, R. Sharma, R. Bhardwaj, R. Bhattacharya, S. Bhattacharya, U. Bhawandeep, D. Bhowmik, S. Dey, S. Dutt, S. Dutta, S. Ghosh, N. Majumdar, K. Mondal, S. Mukhopadhyay, S. Nandan, A. Purohit, P. K. Rout, A. Roy, S. Roy Chowdhury, S. Sarkar, M. Sharan, B. Singh, S. Thakur, P. K. Behera, R. Chudasama, D. Dutta, V. Jha, V. Kumar, A. K. Mohanty, P. K. Netrakanti, L. M. Pant, P. Shukla, A. Topkar, T. Aziz, S. Dugad, B. Mahakud, S. Mitra, G. B. Mohanty, N. Sur, B. Sutar, S. Banerjee, S. Bhattacharya, S. Chatterjee, P. Das, M. Guchait, Sa. Jain, S. Kumar, M. Maity, G. Majumder, K. Mazumdar, N. Sahoo, T. Sarkar, N. Wickramage, S. Chauhan, S. Dube, V. Hegde, A. Kapoor, K. Kothekar, S. Pandey, A. Rane, S. Sharma, S. Chenarani, E. Eskandari Tadavani, S. M. Etesami, M. Khakzad, M. Mohammadi Najafabadi, M. Naseri, S. Paktinat Mehdiabadi, F. Rezaei Hosseinabadi, B. Safarzadeh, M. Zeinali, M. Felcini, M. Grunewald, M. Abbrescia, C. Calabria, A. Colaleo, D. Creanza, L. Cristella, N. De Filippis, M. De Palma, A. Di Florio, F. Errico, L. Fiore, A. Gelmi, G. Iaselli, S. Lezki, G. Maggi, M. Maggi, B. Marangelli, G. Miniello, S. My, S. Nuzzo, A. Pompili, G. Pugliese, R. Radogna, A. Ranieri, G. Selvaggi, A. Sharma, L. Silvestris, R. Venditti, P. Verwilligen, G. Zito, G. Abbiendi, C. Battilana, D. Bonacorsi, L. Borgonovi, S. Braibant-Giacomelli, L. Brigliadori, R. Campanini, P. Capiluppi, A. Castro, F. R. Cavallo, S. S. Chhibra, G. Codispoti, M. Cuffiani, G. M. Dallavalle, F. Fabbri, A. Fanfani, D. Fasanella, P. Giacomelli, C. Grandi, L. Guiducci, S. Marcellini, G. Masetti, A. Montanari, F. L. Navarria, A. Perrotta, A. M. Rossi, T. Rovelli, G. P. Siroli, N. Tosi, S. Albergo, S. Costa, A. Di Mattia, F. Giordano, R. Potenza, A. Tricomi, C. Tuve, G. Barbagli, K. Chatterjee, V. Ciulli, C. Civinini, R. D’Alessandro, E. Focardi, G. Latino, P. Lenzi, M. Meschini, S. Paoletti, L. Russo, G. Sguazzoni, D. Strom, L. Viliani, L. Benussi, S. Bianco, F. Fabbri, D. Piccolo, F. Primavera, V. Calvelli, F. Ferro, F. Ravera, E. Robutti, S. Tosi, A. Benaglia, A. Beschi, L. Brianza, F. Brivio, V. Ciriolo, M. E. Dinardo, S. Fiorendi, S. Gennai, A. Ghezzi, P. Govoni, M. Malberti, S. Malvezzi, R. A. Manzoni, D. Menasce, L. Moroni, M. Paganoni, K. Pauwels, D. Pedrini, S. Pigazzini, S. Ragazzi, T. Tabarelli de Fatis, S. Buontempo, N. Cavallo, S. Di Guida, F. Fabozzi, F. Fienga, G. Galati, A. O. M. Iorio, W. A. Khan, L. Lista, S. Meola, P. Paolucci, C. Sciacca, F. Thyssen, E. Voevodina, P. Azzi, N. Bacchetta, L. Benato, D. Bisello, A. Boletti, R. Carlin, A. Carvalho Antunes De Oliveira, P. Checchia, P. De Castro Manzano, T. Dorigo, U. Dosselli, F. Gasparini, U. Gasparini, A. Gozzelino, S. Lacaprara, M. Margoni, A. T. Meneguzzo, N. Pozzobon, P. Ronchese, R. Rossin, F. Simonetto, A. Tiko, E. Torassa, M. Zanetti, P. Zotto, G. Zumerle, A. Braghieri, A. Magnani, P. Montagna, S. P. Ratti, V. Re, M. Ressegotti, C. Riccardi, P. Salvini, I. Vai, P. Vitulo, L. Alunni Solestizi, M. Biasini, G. M. Bilei, C. Cecchi, D. Ciangottini, L. Fanò, P. Lariccia, R. Leonardi, E. Manoni, G. Mantovani, V. Mariani, M. Menichelli, A. Rossi, A. Santocchia, D. Spiga, K. Androsov, P. Azzurri, G. Bagliesi, L. Bianchini, T. Boccali, L. Borrello, R. Castaldi, M. A. Ciocci, R. Dell’Orso, G. Fedi, L. Giannini, A. Giassi, M. T. Grippo, F. Ligabue, T. Lomtadze, E. Manca, G. Mandorli, A. Messineo, F. Palla, A. Rizzi, P. Spagnolo, R. Tenchini, G. Tonelli, A. Venturi, P. G. Verdini, L. Barone, F. Cavallari, M. Cipriani, N. Daci, D. Del Re, E. Di Marco, M. Diemoz, S. Gelli, E. Longo, B. Marzocchi, P. Meridiani, G. Organtini, F. Pandolfi, R. Paramatti, F. Preiato, S. Rahatlou, C. Rovelli, F. Santanastasio, N. Amapane, R. Arcidiacono, S. Argiro, M. Arneodo, N. Bartosik, R. Bellan, C. Biino, N. Cartiglia, R. Castello, F. Cenna, M. Costa, R. Covarelli, A. Degano, N. Demaria, B. Kiani, C. Mariotti, S. Maselli, E. Migliore, V. Monaco, E. Monteil, M. Monteno, M. M. Obertino, L. Pacher, N. Pastrone, M. Pelliccioni, G. L. Pinna Angioni, A. Romero, M. Ruspa, R. Sacchi, K. Shchelina, V. Sola, A. Solano, A. Staiano, S. Belforte, M. Casarsa, F. Cossutti, G. Della Ricca, A. Zanetti, D. H. Kim, G. N. Kim, M. S. Kim, J. Lee, S. Lee, S. W. Lee, C. S. Moon, Y. D. Oh, S. Sekmen, D. C. Son, Y. C. Yang, H. Kim, D. H. Moon, G. Oh, J. A. Brochero Cifuentes, J. Goh, T. J. Kim, S. Cho, S. Choi, Y. Go, D. Gyun, S. Ha, B. Hong, Y. Jo, Y. Kim, K. Lee, K. S. Lee, S. Lee, J. Lim, S. K. Park, Y. Roh, J. Almond, J. Kim, J. S. Kim, H. Lee, K. Lee, K. Nam, S. B. Oh, B. C. Radburn-Smith, S. h. Seo, U. K. Yang, H. D. Yoo, G. B. Yu, H. Kim, J. H. Kim, J. S. H. Lee, I. C. Park, Y. Choi, C. Hwang, J. Lee, I. Yu, V. Dudenas, A. Juodagalvis, J. Vaitkus, I. Ahmed, Z. A. Ibrahim, M. A. B. Md Ali, F. Mohamad Idris, W. A. T. Wan Abdullah, M. N. Yusli, Z. Zolkapli, M. C. Duran-Osuna, H. Castilla-Valdez, E. De La Cruz-Burelo, G. Ramirez-Sanchez, I. Heredia-De La Cruz, R. I. Rabadan-Trejo, R. Lopez-Fernandez, J. Mejia Guisao, R Reyes-Almanza, A. Sanchez-Hernandez, S. Carrillo Moreno, C. Oropeza Barrera, F. Vazquez Valencia, J. Eysermans, I. Pedraza, H. A. Salazar Ibarguen, C. Uribe Estrada, A. Morelos Pineda, D. Krofcheck, S. Bheesette, P. H. Butler, A. Ahmad, M. Ahmad, Q. Hassan, H. R. Hoorani, A. Saddique, M. A. Shah, M. Shoaib, M. Waqas, H. Bialkowska, M. Bluj, B. Boimska, T. Frueboes, M. Górski, M. Kazana, K. Nawrocki, M. Szleper, P. Traczyk, P. Zalewski, K. Bunkowski, A. Byszuk, K. Doroba, A. Kalinowski, M. Konecki, J. Krolikowski, M. Misiura, M. Olszewski, A. Pyskir, M. Walczak, P. Bargassa, C. Beirão Da Cruz E Silva, A. Di Francesco, P. Faccioli, B. Galinhas, M. Gallinaro, J. Hollar, N. Leonardo, L. Lloret Iglesias, M. V. Nemallapudi, J. Seixas, G. Strong, O. Toldaiev, D. Vadruccio, J. Varela, S. Afanasiev, P. Bunin, M. Gavrilenko, I. Golutvin, I. Gorbunov, A. Kamenev, V. Karjavin, A. Lanev, A. Malakhov, V. Matveev, P. Moisenz, V. Palichik, V. Perelygin, S. Shmatov, S. Shulha, N. Skatchkov, V. Smirnov, N. Voytishin, A. Zarubin, Y. Ivanov, V. Kim, E. Kuznetsova, P. Levchenko, V. Murzin, V. Oreshkin, I. Smirnov, D. Sosnov, V. Sulimov, L. Uvarov, S. Vavilov, A. Vorobyev, Yu. Andreev, A. Dermenev, S. Gninenko, N. Golubev, A. Karneyeu, M. Kirsanov, N. Krasnikov, A. Pashenkov, D. Tlisov, A. Toropin, V. Epshteyn, V. Gavrilov, N. Lychkovskaya, V. Popov, I. Pozdnyakov, G. Safronov, A. Spiridonov, A. Stepennov, V. Stolin, M. Toms, E. Vlasov, A. Zhokin, T. Aushev, A. Bylinkin, R. Chistov, M. Danilov, P. Parygin, D. Philippov, S. Polikarpov, E. Tarkovskii, V. Andreev, M. Azarkin, I. Dremin, M. Kirakosyan, S. V. Rusakov, A. Terkulov, A. Baskakov, A. Belyaev, E. Boos, M. Dubinin, L. Dudko, A. Ershov, A. Gribushin, V. Klyukhin, O. Kodolova, I. Lokhtin, I. Miagkov, S. Obraztsov, S. Petrushanko, V. Savrin, A. Snigirev, V. Blinov, D. Shtol, Y. Skovpen, I. Azhgirey, I. Bayshev, S. Bitioukov, D. Elumakhov, A. Godizov, V. Kachanov, A. Kalinin, D. Konstantinov, P. Mandrik, V. Petrov, R. Ryutin, A. Sobol, S. Troshin, N. Tyurin, A. Uzunian, A. Volkov, A. Babaev, P. Adzic, P. Cirkovic, D. Devetak, M. Dordevic, J. Milosevic, J. Alcaraz Maestre, A. Álvarez Fernández, I. Bachiller, M. Barrio Luna, M. Cerrada, N. Colino, B. De La Cruz, A. Delgado Peris, C. Fernandez Bedoya, J. P. Fernández Ramos, J. Flix, M. C. Fouz, O. Gonzalez Lopez, S. Goy Lopez, J. M. Hernandez, M. I. Josa, D. Moran, A. Pérez-Calero Yzquierdo, J. Puerta Pelayo, I. Redondo, L. Romero, M. S. Soares, A. Triossi, C. Albajar, J. F. de Trocóniz, J. Cuevas, C. Erice, J. Fernandez Menendez, S. Folgueras, I. Gonzalez Caballero, J. R. González Fernández, E. Palencia Cortezon, S. Sanchez Cruz, P. Vischia, J. M. Vizan Garcia, I. J. Cabrillo, A. Calderon, B. Chazin Quero, J. Duarte Campderros, M. Fernandez, P. J. Fernández Manteca, A. García Alonso, J. Garcia-Ferrero, G. Gomez, A. Lopez Virto, J. Marco, C. Martinez Rivero, P. Martinez Ruiz del Arbol, F. Matorras, J. Piedra Gomez, C. Prieels, T. Rodrigo, A. Ruiz-Jimeno, L. Scodellaro, N. Trevisani, I. Vila, R. Vilar Cortabitarte, D. Abbaneo, B. Akgun, E. Auffray, P. Baillon, A. H. Ball, D. Barney, J. Bendavid, M. Bianco, A. Bocci, C. Botta, T. Camporesi, M. Cepeda, G. Cerminara, E. Chapon, Y. Chen, D. d’Enterria, A. Dabrowski, V. Daponte, A. David, M. De Gruttola, A. De Roeck, N. Deelen, M. Dobson, T. du Pree, M. Dünser, N. Dupont, A. Elliott-Peisert, P. Everaerts, F. Fallavollita, G. Franzoni, J. Fulcher, W. Funk, D. Gigi, A. Gilbert, K. Gill, F. Glege, D. Gulhan, J. Hegeman, V. Innocente, A. Jafari, P. Janot, O. Karacheban, J. Kieseler, V. Knünz, A. Kornmayer, M. Krammer, C. Lange, P. Lecoq, C. Lourenço, M. T. Lucchini, L. Malgeri, M. Mannelli, A. Martelli, F. Meijers, J. A. Merlin, S. Mersi, E. Meschi, P. Milenovic, F. Moortgat, M. Mulders, H. Neugebauer, J. Ngadiuba, S. Orfanelli, L. Orsini, F. Pantaleo, L. Pape, E. Perez, M. Peruzzi, A. Petrilli, G. Petrucciani, A. Pfeiffer, M. Pierini, F. M. Pitters, D. Rabady, A. Racz, T. Reis, G. Rolandi, M. Rovere, H. Sakulin, C. Schäfer, C. Schwick, M. Seidel, M. Selvaggi, A. Sharma, P. Silva, P. Sphicas, A. Stakia, J. Steggemann, M. Stoye, M. Tosi, D. Treille, A. Tsirou, V. Veckalns, M. Verweij, W. D. Zeuner, W. Bertl, L. Caminada, K. Deiters, W. Erdmann, R. Horisberger, Q. Ingram, H. C. Kaestli, D. Kotlinski, U. Langenegger, T. Rohe, S. A. Wiederkehr, M. Backhaus, L. Bäni, P. Berger, B. Casal, N. Chernyavskaya, G. Dissertori, M. Dittmar, M. Donegà, C. Dorfer, C. Grab, C. Heidegger, D. Hits, J. Hoss, T. Klijnsma, W. Lustermann, M. Marionneau, M. T. Meinhard, D. Meister, F. Micheli, P. Musella, F. Nessi-Tedaldi, J. Pata, F. Pauss, G. Perrin, L. Perrozzi, M. Quittnat, M. Reichmann, D. Ruini, D. A. Sanz Becerra, M. Schönenberger, L. Shchutska, V. R. Tavolaro, K. Theofilatos, M. L. Vesterbacka Olsson, R. Wallny, D. H. Zhu, T. K. Aarrestad, C. Amsler, D. Brzhechko, M. F. Canelli, A. De Cosa, R. Del Burgo, S. Donato, C. Galloni, T. Hreus, B. Kilminster, I. Neutelings, D. Pinna, G. Rauco, P. Robmann, D. Salerno, K. Schweiger, C. Seitz, Y. Takahashi, A. Zucchetta, V. Candelise, Y. H. Chang, K. y. Cheng, T. H. Doan, Sh. Jain, R. Khurana, C. M. Kuo, W. Lin, A. Pozdnyakov, S. S. Yu, P. Chang, Y. Chao, K. F. Chen, P. H. Chen, F. Fiori, W.-S. Hou, Y. Hsiung, Arun Kumar, Y. F. Liu, R.-S. Lu, E. Paganis, A. Psallidas, A. Steen, J. f. Tsai, B. Asavapibhop, K. Kovitanggoon, G. Singh, N. Srimanobhas, A. Bat, F. Boran, S. Cerci, S. Damarseckin, Z. S. Demiroglu, C. Dozen, I. Dumanoglu, S. Girgis, G. Gokbulut, Y. Guler, I. Hos, E. E. Kangal, O. Kara, A. Kayis Topaksu, U. Kiminsu, M. Oglakci, G. Onengut, K. Ozdemir, D. Sunar Cerci, U. G. Tok, H. Topakli, S. Turkcapar, I. S. Zorbakir, C. Zorbilmez, G. Karapinar, K. Ocalan, M. Yalvac, M. Zeyrek, I. O. Atakisi, E. Gülmez, M. Kaya, O. Kaya, S. Tekten, E. A. Yetkin, M. N. Agaras, S. Atay, A. Cakir, K. Cankocak, Y. Komurcu, B. Grynyov, L. Levchuk, F. Ball, L. Beck, J. J. Brooke, D. Burns, E. Clement, D. Cussans, O. Davignon, H. Flacher, J. Goldstein, G. P. Heath, H. F. Heath, L. Kreczko, D. M. Newbold, S. Paramesvaran, T. Sakuma, S. Seif El Nasr-storey, D. Smith, V. J. Smith, K. W. Bell, A. Belyaev, C. Brew, R. M. Brown, D. Cieri, D. J. A. Cockerill, J. A. Coughlan, K. Harder, S. Harper, J. Linacre, E. Olaiya, D. Petyt, C. H. Shepherd-Themistocleous, A. Thea, I. R. Tomalin, T. Williams, W. J. Womersley, G. Auzinger, R. Bainbridge, P. Bloch, J. Borg, S. Breeze, O. Buchmuller, A. Bundock, S. Casasso, D. Colling, L. Corpe, P. Dauncey, G. Davies, M. Della Negra, R. Di Maria, Y. Haddad, G. Hall, G. Iles, T. James, M. Komm, R. Lane, C. Laner, L. Lyons, A.-M. Magnan, S. Malik, L. Mastrolorenzo, T. Matsushita, J. Nash, A. Nikitenko, V. Palladino, M. Pesaresi, A. Richards, A. Rose, E. Scott, C. Seez, A. Shtipliyski, T. Strebler, S. Summers, A. Tapper, K. Uchida, M. Vazquez Acosta, T. Virdee, N. Wardle, D. Winterbottom, J. Wright, S. C. Zenz, J. E. Cole, P. R. Hobson, A. Khan, P. Kyberd, A. Morton, I. D. Reid, L. Teodorescu, S. Zahid, A. Borzou, K. Call, J. Dittmann, K. Hatakeyama, H. Liu, N. Pastika, C. Smith, R. Bartek, A. Dominguez, A. Buccilli, S. I. Cooper, C. Henderson, P. Rumerio, C. West, D. Arcaro, A. Avetisyan, T. Bose, D. Gastler, D. Rankin, C. Richardson, J. Rohlf, L. Sulak, D. Zou, G. Benelli, D. Cutts, M. Hadley, J. Hakala, U. Heintz, J. M. Hogan, K. H. M. Kwok, E. Laird, G. Landsberg, J. Lee, Z. Mao, M. Narain, J. Pazzini, S. Piperov, S. Sagir, R. Syarif, D. Yu, R. Band, C. Brainerd, R. Breedon, D. Burns, M. Calderon De La Barca Sanchez, M. Chertok, J. Conway, R. Conway, P. T. Cox, R. Erbacher, C. Flores, G. Funk, W. Ko, R. Lander, C. Mclean, M. Mulhearn, D. Pellett, J. Pilot, S. Shalhout, M. Shi, J. Smith, D. Stolp, D. Taylor, K. Tos, M. Tripathi, Z. Wang, F. Zhang, M. Bachtis, C. Bravo, R. Cousins, A. Dasgupta, A. Florent, J. Hauser, M. Ignatenko, N. Mccoll, S. Regnard, D. Saltzberg, C. Schnaible, V. Valuev, E. Bouvier, K. Burt, R. Clare, J. Ellison, J. W. Gary, S. M. A. Ghiasi Shirazi, G. Hanson, G. Karapostoli, E. Kennedy, F. Lacroix, O. R. Long, M. Olmedo Negrete, M. I. Paneva, W. Si, L. Wang, H. Wei, S. Wimpenny, B. R. Yates, J. G. Branson, S. Cittolin, M. Derdzinski, R. Gerosa, D. Gilbert, B. Hashemi, A. Holzner, D. Klein, G. Kole, V. Krutelyov, J. Letts, M. Masciovecchio, D. Olivito, S. Padhi, M. Pieri, M. Sani, V. Sharma, S. Simon, M. Tadel, A. Vartak, S. Wasserbaech, J. Wood, F. Würthwein, A. Yagil, G. Zevi Della Porta, N. Amin, R. Bhandari, J. Bradmiller-Feld, C. Campagnari, M. Citron, A. Dishaw, V. Dutta, M. Franco Sevilla, L. Gouskos, R. Heller, J. Incandela, A. Ovcharova, H. Qu, J. Richman, D. Stuart, I. Suarez, J. Yoo, D. Anderson, A. Bornheim, J. Bunn, J. M. Lawhorn, H. B. Newman, T. Q. Nguyen, C. Pena, M. Spiropulu, J. R. Vlimant, R. Wilkinson, S. Xie, Z. Zhang, R. Y. Zhu, M. B. Andrews, T. Ferguson, T. Mudholkar, M. Paulini, J. Russ, M. Sun, H. Vogel, I. Vorobiev, M. Weinberg, J. P. Cumalat, W. T. Ford, F. Jensen, A. Johnson, M. Krohn, S. Leontsinis, E. MacDonald, T. Mulholland, K. Stenson, K. A. Ulmer, S. R. Wagner, J. Alexander, J. Chaves, Y. Cheng, J. Chu, A. Datta, K. Mcdermott, N. Mirman, J. R. Patterson, D. Quach, A. Rinkevicius, A. Ryd, L. Skinnari, L. Soffi, S. M. Tan, Z. Tao, J. Thom, J. Tucker, P. Wittich, M. Zientek, S. Abdullin, M. Albrow, M. Alyari, G. Apollinari, A. Apresyan, A. Apyan, S. Banerjee, L. A. T. Bauerdick, A. Beretvas, J. Berryhill, P. C. Bhat, G. Bolla, K. Burkett, J. N. Butler, A. Canepa, G. B. Cerati, H. W. K. Cheung, F. Chlebana, M. Cremonesi, J. Duarte, V. D. Elvira, J. Freeman, Z. Gecse, E. Gottschalk, L. Gray, D. Green, S. Grünendahl, O. Gutsche, J. Hanlon, R. M. Harris, S. Hasegawa, J. Hirschauer, Z. Hu, B. Jayatilaka, S. Jindariani, M. Johnson, U. Joshi, B. Klima, M. J. Kortelainen, B. Kreis, S. Lammel, D. Lincoln, R. Lipton, M. Liu, T. Liu, R. Lopes De Sá, J. Lykken, K. Maeshima, N. Magini, J. M. Marraffino, D. Mason, P. McBride, P. Merkel, S. Mrenna, S. Nahn, V. O’Dell, K. Pedro, O. Prokofyev, G. Rakness, L. Ristori, A. Savoy-Navarro, B. Schneider, E. Sexton-Kennedy, A. Soha, W. J. Spalding, L. Spiegel, S. Stoynev, J. Strait, N. Strobbe, L. Taylor, S. Tkaczyk, N. V. Tran, L. Uplegger, E. W. Vaandering, C. Vernieri, M. Verzocchi, R. Vidal, M. Wang, H. A. Weber, A. Whitbeck, W. Wu, D. Acosta, P. Avery, P. Bortignon, D. Bourilkov, A. Brinkerhoff, A. Carnes, M. Carver, D. Curry, R. D. Field, I. K. Furic, S. V. Gleyzer, B. M. Joshi, J. Konigsberg, A. Korytov, K. Kotov, P. Ma, K. Matchev, H. Mei, G. Mitselmakher, K. Shi, D. Sperka, N. Terentyev, L. Thomas, J. Wang, S. Wang, J. Yelton, Y. R. Joshi, S. Linn, P. Markowitz, J. L. Rodriguez, A. Ackert, T. Adams, A. Askew, S. Hagopian, V. Hagopian, K. F. Johnson, T. Kolberg, G. Martinez, T. Perry, H. Prosper, A. Saha, A. Santra, V. Sharma, R. Yohay, M. M. Baarmand, V. Bhopatkar, S. Colafranceschi, M. Hohlmann, D. Noonan, T. Roy, F. Yumiceva, M. R. Adams, L. Apanasevich, D. Berry, R. R. Betts, R. Cavanaugh, X. Chen, S. Dittmer, O. Evdokimov, C. E. Gerber, D. A. Hangal, D. J. Hofman, K. Jung, J. Kamin, I. D. Sandoval Gonzalez, M. B. Tonjes, N. Varelas, H. Wang, Z. Wu, J. Zhang, B. Bilki, W. Clarida, K. Dilsiz, S. Durgut, R. P. Gandrajula, M. Haytmyradov, V. Khristenko, J.-P. Merlo, H. Mermerkaya, A. Mestvirishvili, A. Moeller, J. Nachtman, H. Ogul, Y. Onel, F. Ozok, A. Penzo, C. Snyder, E. Tiras, J. Wetzel, K. Yi, B. Blumenfeld, A. Cocoros, N. Eminizer, D. Fehling, L. Feng, A. V. Gritsan, W. T. Hung, P. Maksimovic, J. Roskes, U. Sarica, M. Swartz, M. Xiao, C. You, A. Al-bataineh, P. Baringer, A. Bean, S. Boren, J. Bowen, J. Castle, S. Khalil, A. Kropivnitskaya, D. Majumder, W. Mcbrayer, M. Murray, C. Rogan, C. Royon, S. Sanders, E. Schmitz, J. D. Tapia Takaki, Q. Wang, A. Ivanov, K. Kaadze, Y. Maravin, A. Modak, A. Mohammadi, L. K. Saini, N. Skhirtladze, F. Rebassoo, D. Wright, A. Baden, O. Baron, A. Belloni, S. C. Eno, Y. Feng, C. Ferraioli, N. J. Hadley, S. Jabeen, G. Y. Jeng, R. G. Kellogg, J. Kunkle, A. C. Mignerey, F. Ricci-Tam, Y. H. Shin, A. Skuja, S. C. Tonwar, D. Abercrombie, B. Allen, V. Azzolini, R. Barbieri, A. Baty, G. Bauer, R. Bi, S. Brandt, W. Busza, I. A. Cali, M. D’Alfonso, Z. Demiragli, G. Gomez Ceballos, M. Goncharov, P. Harris, D. Hsu, M. Hu, Y. Iiyama, G. M. Innocenti, M. Klute, D. Kovalskyi, Y.-J. Lee, A. Levin, P. D. Luckey, B. Maier, A. C. Marini, C. Mcginn, C. Mironov, S. Narayanan, X. Niu, C. Paus, C. Roland, G. Roland, G. S. F. Stephans, K. Sumorok, K. Tatar, D. Velicanu, J. Wang, T. W. Wang, B. Wyslouch, S. Zhaozhong, A. C. Benvenuti, R. M. Chatterjee, A. Evans, P. Hansen, S. Kalafut, Y. Kubota, Z. Lesko, J. Mans, S. Nourbakhsh, N. Ruckstuhl, R. Rusack, J. Turkewitz, M. A. Wadud, J. G. Acosta, S. Oliveros, E. Avdeeva, K. Bloom, D. R. Claes, C. Fangmeier, F. Golf, R. Gonzalez Suarez, R. Kamalieddin, I. Kravchenko, J. Monroy, J. E. Siado, G. R. Snow, B. Stieger, A. Godshalk, C. Harrington, I. Iashvili, D. Nguyen, A. Parker, S. Rappoccio, B. Roozbahani, G. Alverson, E. Barberis, C. Freer, A. Hortiangtham, A. Massironi, D. M. Morse, T. Orimoto, R. Teixeira De Lima, T. Wamorkar, B. Wang, A. Wisecarver, D. Wood, S. Bhattacharya, O. Charaf, K. A. Hahn, N. Mucia, N. Odell, M. H. Schmitt, K. Sung, M. Trovato, M. Velasco, R. Bucci, N. Dev, M. Hildreth, K. Hurtado Anampa, C. Jessop, D. J. Karmgard, N. Kellams, K. Lannon, W. Li, N. Loukas, N. Marinelli, F. Meng, C. Mueller, Y. Musienko, M. Planer, A. Reinsvold, R. Ruchti, P. Siddireddy, G. Smith, S. Taroni, M. Wayne, A. Wightman, M. Wolf, A. Woodard, J. Alimena, L. Antonelli, B. Bylsma, L. S. Durkin, S. Flowers, B. Francis, A. Hart, C. Hill, W. Ji, T. Y. Ling, W. Luo, B. L. Winer, H. W. Wulsin, S. Cooperstein, O. Driga, P. Elmer, J. Hardenbrook, P. Hebda, S. Higginbotham, A. Kalogeropoulos, D. Lange, J. Luo, D. Marlow, K. Mei, I. Ojalvo, J. Olsen, C. Palmer, P. Piroué, J. Salfeld-Nebgen, D. Stickland, C. Tully, S. Malik, S. Norberg, A. Barker, V. E. Barnes, S. Das, L. Gutay, M. Jones, A. W. Jung, A. Khatiwada, D. H. Miller, N. Neumeister, C. C. Peng, H. Qiu, J. F. Schulte, J. Sun, F. Wang, R. Xiao, W. Xie, T. Cheng, J. Dolen, N. Parashar, Z. Chen, K. M. Ecklund, S. Freed, F. J. M. Geurts, M. Guilbaud, M. Kilpatrick, W. Li, B. Michlin, B. P. Padley, J. Roberts, J. Rorie, W. Shi, Z. Tu, J. Zabel, A. Zhang, A. Bodek, P. de Barbaro, R. Demina, Y. t. Duh, T. Ferbel, M. Galanti, A. Garcia-Bellido, J. Han, O. Hindrichs, A. Khukhunaishvili, K. H. Lo, P. Tan, M. Verzetti, R. Ciesielski, K. Goulianos, C. Mesropian, A. Agapitos, J. P. Chou, Y. Gershtein, T. A. Gómez Espinosa, E. Halkiadakis, M. Heindl, E. Hughes, S. Kaplan, R. Kunnawalkam Elayavalli, S. Kyriacou, A. Lath, R. Montalvo, K. Nash, M. Osherson, H. Saka, S. Salur, S. Schnetzer, D. Sheffield, S. Somalwar, R. Stone, S. Thomas, P. Thomassen, M. Walker, A. G. Delannoy, J. Heideman, G. Riley, K. Rose, S. Spanier, K. Thapa, O. Bouhali, A. Castaneda Hernandez, A. Celik, M. Dalchenko, M. De Mattia, A. Delgado, S. Dildick, R. Eusebi, J. Gilmore, T. Huang, T. Kamon, R. Mueller, Y. Pakhotin, R. Patel, A. Perloff, L. Perniè, D. Rathjens, A. Safonov, A. Tatarinov, N. Akchurin, J. Damgov, F. De Guio, P. R. Dudero, J. Faulkner, E. Gurpinar, S. Kunori, K. Lamichhane, S. W. Lee, T. Mengke, S. Muthumuni, T. Peltola, S. Undleeb, I. Volobouev, Z. Wang, S. Greene, A. Gurrola, R. Janjam, W. Johns, C. Maguire, A. Melo, H. Ni, K. Padeken, J. D. Ruiz Alvarez, P. Sheldon, S. Tuo, J. Velkovska, Q. Xu, M. W. Arenton, P. Barria, B. Cox, R. Hirosky, M. Joyce, A. Ledovskoy, H. Li, C. Neu, T. Sinthuprasith, Y. Wang, E. Wolfe, F. Xia, R. Harr, P. E. Karchin, N. Poudyal, J. Sturdy, P. Thapa, S. Zaleski, M. Brodski, J. Buchanan, C. Caillol, D. Carlsmith, S. Dasu, L. Dodd, S. Duric, B. Gomber, M. Grothe, M. Herndon, A. Hervé, U. Hussain, P. Klabbers, A. Lanaro, A. Levine, K. Long, R. Loveless, V. Rekovic, T. Ruggles, A. Savin, N. Smith, W. H. Smith, N. Woods, CMS Collaboration

**Affiliations:** 1 Yerevan Physics Institute, Yerevan, Armenia; 2 Institut für Hochenergiephysik, Vienna, Austria; 3 Institute for Nuclear Problems, Minsk, Belarus; 4 Universiteit Antwerpen, Antwerp, Belgium; 5 Vrije Universiteit Brussel, Brussels, Belgium; 6 Université Libre de Bruxelles, Brussels, Belgium; 7 Ghent University, Ghent, Belgium; 8 Université Catholique de Louvain, Louvain-la-Neuve, Belgium; 9 Centro Brasileiro de Pesquisas Fisicas, Rio de Janeiro, Brazil; 10 Universidade do Estado do Rio de Janeiro, Rio de Janeiro, Brazil; 11 Universidade Estadual Paulista, Universidade Federal do ABC, São Paulo, Brazil; 12 Institute for Nuclear Research and Nuclear Energy, Bulgarian Academy of Sciences, Sofia, Bulgaria; 13 University of Sofia, Sofia, Bulgaria; 14 Beihang University, Beijing, China; 15 Institute of High Energy Physics, Beijing, China; 16 State Key Laboratory of Nuclear Physics and Technology, Peking University, Beijing, China; 17 Tsinghua University, Beijing, China; 18 Universidad de Los Andes, Bogotá, Colombia; 19 University of Split, Faculty of Electrical Engineering, Mechanical Engineering and Naval Architecture, Split, Croatia; 20 University of Split, Faculty of Science, Split, Croatia; 21 Institute Rudjer Boskovic, Zagreb, Croatia; 22 University of Cyprus, Nicosia, Cyprus; 23 Charles University, Prague, Czech Republic; 24 Universidad San Francisco de Quito, Quito, Ecuador; 25 Academy of Scientific Research and Technology of the Arab Republic of Egypt, Egyptian Network of High Energy Physics, Cairo, Egypt; 26 National Institute of Chemical Physics and Biophysics, Tallinn, Estonia; 27 Department of Physics, University of Helsinki, Helsinki, Finland; 28 Helsinki Institute of Physics, Helsinki, Finland; 29 Lappeenranta University of Technology, Lappeenranta, Finland; 30 IRFU, CEA, Université Paris-Saclay, Gif-sur-Yvette, France; 31 Laboratoire Leprince-Ringuet, Ecole polytechnique, CNRS/IN2P3, Université Paris-Saclay, Palaiseau, France; 32 Université de Strasbourg, CNRS, IPHC UMR 7178, 67000 Strasbourg, France; 33 Centre de Calcul de l’Institut National de Physique Nucleaire et de Physique des Particules, CNRS/IN2P3, Villeurbanne, France; 34 Université de Lyon, Université Claude Bernard Lyon 1, CNRS-IN2P3, Institut de Physique Nucléaire de Lyon, Villeurbanne, France; 35 Georgian Technical University, Tbilisi, Georgia; 36 Tbilisi State University, Tbilisi, Georgia; 37 RWTH Aachen University, I. Physikalisches Institut, Aachen, Germany; 38 RWTH Aachen University, III. Physikalisches Institut A, Aachen, Germany; 39 RWTH Aachen University, III. Physikalisches Institut B, Aachen, Germany; 40 Deutsches Elektronen-Synchrotron, Hamburg, Germany; 41 University of Hamburg, Hamburg, Germany; 42 Institut für Experimentelle Teilchenphysik, Karlsruhe, Germany; 43 Institute of Nuclear and Particle Physics (INPP), NCSR Demokritos, Agia Paraskevi, Greece; 44 National and Kapodistrian University of Athens, Athens, Greece; 45 National Technical University of Athens, Athens, Greece; 46 University of Ioánnina, Ioannina, Greece; 47 MTA-ELTE Lendület CMS Particle and Nuclear Physics Group, Eötvös Loránd University, Budapest, Hungary; 48 Wigner Research Centre for Physics, Budapest, Hungary; 49 Institute of Nuclear Research ATOMKI, Debrecen, Hungary; 50 Institute of Physics, University of Debrecen, Debrecen, Hungary; 51 Indian Institute of Science (IISc), Bangalore, India; 52 National Institute of Science Education and Research, Bhubaneswar, India; 53 Panjab University, Chandigarh, India; 54 University of Delhi, Delhi, India; 55 Saha Institute of Nuclear Physics, HBNI, Kolkata, India; 56 Indian Institute of Technology Madras, Madras, India; 57 Bhabha Atomic Research Centre, Mumbai, India; 58 Tata Institute of Fundamental Research-A, Mumbai, India; 59 Tata Institute of Fundamental Research-B, Mumbai, India; 60 Indian Institute of Science Education and Research (IISER), Pune, India; 61 Institute for Research in Fundamental Sciences (IPM), Tehran, Iran; 62 University College Dublin, Dublin, Ireland; 63 INFN Sezione di Bari, Università di Bari, Politecnico di Bari, Bari, Italy; 64 INFN Sezione di Bologna, Università di Bologna, Bologna, Italy; 65 INFN Sezione di Catania, Università di Catania, Catania, Italy; 66 INFN Sezione di Firenze, Università di Firenze, Florence, Italy; 67 INFN Laboratori Nazionali di Frascati, Frascati, Italy; 68 INFN Sezione di Genova, Università di Genova, Genoa, Italy; 69 INFN Sezione di Milano-Bicocca, Università di Milano-Bicocca, Milan, Italy; 70 INFN Sezione di Napoli, Università di Napoli ’Federico II’ , Napoli, Italy, Università della Basilicata, Potenza, Italy, Università G. Marconi, Rome, Italy; 71 INFN Sezione di Padova, Università di Padova, Padova, Italy, Università di Trento, Trento, Italy; 72 INFN Sezione di Pavia, Università di Pavia, Pavia, Italy; 73 INFN Sezione di Perugia, Università di Perugia, Perugia, Italy; 74 INFN Sezione di Pisa, Università di Pisa, Scuola Normale Superiore di Pisa, Pisa, Italy; 75 INFN Sezione di Roma, Sapienza Università di Roma, Rome, Italy; 76 INFN Sezione di Torino, Università di Torino, Torino, Italy, Università del Piemonte Orientale, Novara, Italy; 77 INFN Sezione di Trieste, Università di Trieste, Trieste, Italy; 78 Kyungpook National University, Daegu, Korea; 79 Chonnam National University, Institute for Universe and Elementary Particles, Kwangju, Korea; 80 Hanyang University, Seoul, Korea; 81 Korea University, Seoul, Korea; 82 Seoul National University, Seoul, Korea; 83 University of Seoul, Seoul, Korea; 84 Sungkyunkwan University, Suwon, Korea; 85 Vilnius University, Vilnius, Lithuania; 86 National Centre for Particle Physics, Universiti Malaya, Kuala Lumpur, Malaysia; 87 Centro de Investigacion y de Estudios Avanzados del IPN, Mexico City, Mexico; 88 Universidad Iberoamericana, Mexico City, Mexico; 89 Benemerita Universidad Autonoma de Puebla, Puebla, Mexico; 90 Universidad Autónoma de San Luis Potosí, San Luis Potosí, Mexico; 91 University of Auckland, Auckland, New Zealand; 92 University of Canterbury, Christchurch, New Zealand; 93 National Centre for Physics, Quaid-I-Azam University, Islamabad, Pakistan; 94 National Centre for Nuclear Research, Swierk, Poland; 95 Institute of Experimental Physics, Faculty of Physics, University of Warsaw, Warsaw, Poland; 96 Laboratório de Instrumentação e Física Experimental de Partículas, Lisbon, Portugal; 97 Joint Institute for Nuclear Research, Dubna, Russia; 98 Petersburg Nuclear Physics Institute, Gatchina, St. Petersburg, Russia; 99 Institute for Nuclear Research, Moscow, Russia; 100 Institute for Theoretical and Experimental Physics, Moscow, Russia; 101 Moscow Institute of Physics and Technology, Moscow, Russia; 102 National Research Nuclear University ‘Moscow Engineering Physics Institute’ (MEPhI), Moscow, Russia; 103 P.N. Lebedev Physical Institute, Moscow, Russia; 104 Skobeltsyn Institute of Nuclear Physics, Lomonosov Moscow State University, Moscow, Russia; 105 Novosibirsk State University (NSU), Novosibirsk, Russia; 106 State Research Center of Russian Federation, Institute for High Energy Physics of NRC, Kurchatov Institute, Protvino, Russia; 107 National Research Tomsk Polytechnic University, Tomsk, Russia; 108 University of Belgrade, Faculty of Physics and Vinca Institute of Nuclear Sciences, Belgrade, Serbia; 109 Centro de Investigaciones Energéticas Medioambientales y Tecnológicas (CIEMAT), Madrid, Spain; 110 Universidad Autónoma de Madrid, Madrid, Spain; 111 Universidad de Oviedo, Oviedo, Spain; 112 Instituto de Física de Cantabria (IFCA), CSIC-Universidad de Cantabria, Santander, Spain; 113 CERN, European Organization for Nuclear Research, Geneva, Switzerland; 114 Paul Scherrer Institut, Villigen, Switzerland; 115 ETH Zurich, Institute for Particle Physics and Astrophysics (IPA), Zurich, Switzerland; 116 Universität Zürich, Zurich, Switzerland; 117 National Central University, Chung-Li, Taiwan; 118 National Taiwan University (NTU), Taipei, Taiwan; 119 Department of Physics, Faculty of Science, Chulalongkorn University, Bangkok, Thailand; 120 Physics Department, Science and Art Faculty, Çukurova University, Adana, Turkey; 121 Physics Department, Middle East Technical University, Ankara, Turkey; 122 Bogazici University, Istanbul, Turkey; 123 Istanbul Technical University, Istanbul, Turkey; 124 Institute for Scintillation Materials of National Academy of Science of Ukraine, Kharkov, Ukraine; 125 National Scientific Center, Kharkov Institute of Physics and Technology, Kharkov, Ukraine; 126 University of Bristol, Bristol, UK; 127 Rutherford Appleton Laboratory, Didcot, UK; 128 Imperial College, London, UK; 129 Brunel University, Uxbridge, UK; 130 Baylor University, Waco, USA; 131 Catholic University of America, Washington, DC USA; 132 The University of Alabama, Tuscaloosa, USA; 133 Boston University, Boston, USA; 134 Brown University, Providence, USA; 135 University of California, Davis, Davis, USA; 136 University of California, Los Angeles, USA; 137 University of California, Riverside, Riverside, USA; 138 University of California, San Diego, La Jolla, USA; 139 Department of Physics, University of California, Santa Barbara, Santa Barbara, USA; 140 California Institute of Technology, Pasadena, USA; 141 Carnegie Mellon University, Pittsburgh, USA; 142 University of Colorado Boulder, Boulder, USA; 143 Cornell University, Ithaca, USA; 144 Fermi National Accelerator Laboratory, Batavia, USA; 145 University of Florida, Gainesville, USA; 146 Florida International University, Miami, USA; 147 Florida State University, Tallahassee, USA; 148 Florida Institute of Technology, Melbourne, USA; 149 University of Illinois at Chicago (UIC), Chicago, USA; 150 The University of Iowa, Iowa City, USA; 151 Johns Hopkins University, Baltimore, USA; 152 The University of Kansas, Lawrence, USA; 153 Kansas State University, Manhattan, USA; 154 Lawrence Livermore National Laboratory, Livermore, USA; 155 University of Maryland, College Park, USA; 156 Massachusetts Institute of Technology, Cambridge, USA; 157 University of Minnesota, Minneapolis, USA; 158 University of Mississippi, Oxford, USA; 159 University of Nebraska-Lincoln, Lincoln, USA; 160 State University of New York at Buffalo, Buffalo, USA; 161 Northeastern University, Boston, USA; 162 Northwestern University, Evanston, USA; 163 University of Notre Dame, Notre Dame, USA; 164 The Ohio State University, Columbus, USA; 165 Princeton University, Princeton, USA; 166 University of Puerto Rico, Mayagüez, USA; 167 Purdue University, West Lafayette, USA; 168 Purdue University Northwest, Hammond, USA; 169 Rice University, Houston, USA; 170 University of Rochester, Rochester, USA; 171 The Rockefeller University, New York, USA; 172 Rutgers, The State University of New Jersey, Piscataway, USA; 173 University of Tennessee, Knoxville, USA; 174 Texas A&M University, College Station, USA; 175 Texas Tech University, Lubbock, USA; 176 Vanderbilt University, Nashville, USA; 177 University of Virginia, Charlottesville, USA; 178 Wayne State University, Detroit, USA; 179 University of Wisconsin-Madison, Madison, WI USA; 180 CERN, 1211 Geneva 23, Switzerland

## Abstract

A search is presented for physics beyond the standard model, based on measurements of dijet angular distributions in proton–proton collisions at $\sqrt{s}=13\hbox {TeV}$. The data collected with the CMS detector at the LHC correspond to an integrated luminosity of 35.9$\,\text {fb}^{-1}$. The observed distributions, corrected to particle level, are found to be in agreement with predictions from perturbative quantum chromodynamics that include electroweak corrections. Constraints are placed on models containing quark contact interactions, extra spatial dimensions, quantum black holes, or dark matter, using the detector-level distributions. In a benchmark model where only left-handed quarks participate, contact interactions are excluded at the 95% confidence level up to a scale of 12.8 or 17.5TeV, for destructive or constructive interference, respectively. The most stringent lower limits to date are set on the ultraviolet cutoff in the Arkani–Hamed–Dimopoulos–Dvali model of extra dimensions. In the Giudice–Rattazzi–Wells convention, the cutoff scale is excluded up to 10.1TeV. The production of quantum black holes is excluded for masses below 5.9 and 8.2TeV, depending on the model. For the first time, lower limits between 2.0 and 4.6TeVare set on the mass of a dark matter mediator for (axial-)vector mediators, for the universal quark coupling $g_{\mathrm {\mathrm {q}}} =1.0$.

## Introduction

Pairs of highly energetic jets (dijets) are produced at high rates in proton–proton collisions at the CERN LHC through pointlike scattering of quarks and gluons. Despite its enormous success, the shortcomings of the standard model (SM) are well known. Many theories of physics beyond the standard model (BSM) that alter the interaction of quarks and gluons from that predicted by perturbative quantum chromodynamics (QCD) give rise to narrow or wide resonances or even to nonresonant dijet signatures. Examples that have received widespread attention include models with dark matter (DM) [1–5], quark compositeness [6–8], extra spatial dimensions [9, 10], and quantum black holes [11–15]. Resonances with an intrinsic width of the order of the experimental resolution can be constrained by searches in the dijet invariant mass spectrum [16–18]. These searches, however, are not very sensitive to wide resonances or nonresonant signatures; a more effective strategy to constrain such signatures is the study of dijet angular distributions [19].

The angular distribution of dijets relative to the beam direction is sensitive to the dynamics of the scattering process. Furthermore, since the angular distributions of the dominant underlying QCD processes of $\mathrm {q}\mathrm {g}\rightarrow \mathrm {q}\mathrm {g}$, $\mathrm {q}\mathrm {q}' \rightarrow \mathrm {q}\mathrm {q}'$, $\mathrm {q}\mathrm {q}\rightarrow \mathrm {q}\mathrm {q}$, $\mathrm {g}\mathrm {g}\rightarrow \mathrm {g}\mathrm {g}$, are all similar [20], the dijet angular distribution is insensitive to uncertainties in the parton distribution functions (PDFs). The dijet angular distribution is typically expressed in terms of $\chi _{\text {dijet}} = \exp (|y_1 -y_2 |)$, where $y_1$ and $y_2$ are the rapidities of the two jets with the highest transverse momentum $p_{\mathrm {T}}$ (the leading jets). For collinear massless parton scattering, $\chi _{\text {dijet}}$ takes the form $\chi _{\text {dijet}} =(1+|\cos \theta ^{*} |)/(1-|\cos \theta ^{*} |)$, where $\theta ^{*}$ is the polar scattering angle in the parton-parton center-of-mass (CM) frame. The choice of $\chi _{\text {dijet}}$, rather than $\theta ^{*}$, to measure the dijet angular distribution is motivated by the fact that in Rutherford scattering, where only *t*-channel scattering contributes to the partonic cross section, the $\chi _{\text {dijet}}$ distribution is independent of $|y_1 -y_2 |$ [20]. In contrast, BSM processes may have scattering angle distributions that are closer to being isotropic than those given by QCD processes and can be identified by an excess of events at small values of $\chi _{\text {dijet}}$. Previous measurements of dijet angular distributions at the LHC have been reported by the ATLAS [17, 21–25] and CMS [26–29] Collaborations.

In a simplified model of interactions between DM particles and quarks  [1–4, 30, 31], the spin-1 (vector or axial-vector) DM mediator particle with unknown mass $M_{\text {Med}}$ is assumed to decay only to pairs of quarks or pairs of DM particles, with mass $m_{\mathrm {DM}}$, and with a universal quark coupling $g_{\mathrm {\mathrm {q}}}$ and a DM coupling $g_{\mathrm {DM}}$. In this model, the relative width of the DM mediator increases monotonically with increasing $g_{\mathrm {\mathrm {q}}}$. In a scenario where $g_{\mathrm {\mathrm {q}}} =0.25$ and in which the relative widths for vector and axial-vector mediators in the dark matter decay channels are negligible, values of $M_{\text {Med}}$ below 3.0TeVwere excluded by narrow dijet resonance searches [17, 18]. A search for narrow and broad dijet resonances set constraints on mediator widths up to 30% ($g_{\mathrm {\mathrm {q}}} <0.75$) and masses up to 4 TeV [32]. Searches for invisible particles produced in association with quarks or bosons [33–35] have excluded vector and axial-vector mediators below 1.8 (2.1)TeVfor $g_{\mathrm {\mathrm {q}}} =0.25$ ($g_{\mathrm {\mathrm {q}}} =1.0$) and $g_{\mathrm {DM}} =1.0$ [34].

A common signature of quark compositeness [6–8], at energies well below the characteristic mass scale $\varLambda$ for new interactions between quark constituents, is the four-fermion contact interaction (CI). The most stringent limits on quark CIs come from searches in dijet angular distributions at large dijet invariant masses ($M_{\mathrm {jj}}$) [17, 29], and in inclusive jet $p_{\mathrm {T}}$ distributions [36]. The publication from the ATLAS Collaboration [17] provides lower limits on the quark CI scales from 13.1 to 29.5TeV, depending on the details of the model.

The Arkani–Hamed–Dimopoulos–Dvali (ADD) model [9, 10] of compactified large extra dimensions (EDs) provides a possible solution to the hierarchy problem of the standard model. It predicts signatures of virtual graviton exchange that result in a nonresonant enhancement of dijet production in proton–proton collisions, whose angular distribution differs from the predictions of QCD. Signatures from virtual graviton exchange have previously been sought at the LHC in various final states, where the most stringent limits arise from the CMS search with dijet angular distributions [29], which excludes the ultraviolet cutoff in the ADD framework up to 7.9–11.2TeV, depending on the parameterization of the model.

In models with large EDs, the fundamental Planck scale (${M_\mathrm {Pl}}$) is assumed to be closer to the electroweak (EW) scale, thereby allowing black hole production at the LHC [11–15]. Semiclassical black holes, which have mass much larger than ${M_\mathrm {Pl}}$, decay into multiple jets through Hawking radiation [37]. Quantum black holes (QBHs), which are produced with mass close to ${M_\mathrm {Pl}}$, decay predominantly into dijets and can be studied using dijet angular distributions [38–40]. Recent searches for QBHs with dijet final states at the LHC reported in Refs. [17, 29] exclude QBHs with masses below 8.9TeV.

In this paper, we present a search for new physics, specifically DM mediators, CIs, EDs, and QBHs, using measurements of dijet angular distributions. The signature of the signals can be categorized into nonresonant excesses at high $M_{\mathrm {jj}}$ as predicted by the CI and ADD models and resonances from the decay of QBHs and DM mediators that could appear across the whole range of the $M_{\mathrm {jj}}$ spectrum. The searches are performed by comparing detector-level dijet angular distributions with BSM predictions that have been adjusted to include detector resolution effects. This eliminates some systematic uncertainties that are introduced when correcting the dijet angular distributions for detector effects and simplifies the statistical evaluation. The dijet angular distributions are also corrected to particle level to facilitate comparisons with other theoretical predictions and published in HEPData.

## The CMS detector

The CMS apparatus is based on a superconducting solenoid of 6$\text {\,m}$ internal diameter, providing an axial field of 3.8$\text {\,T}$. Within the solenoid and nearest to the interaction point are the silicon pixel and strip trackers. Surrounding the tracker volume are the lead tungstate crystal electromagnetic calorimeter and the brass and scintillator hadron calorimeter. The trackers cover a pseudorapidity region of $|\eta | < 2.5$ while the calorimeters cover $|\eta | < 3.0$. In addition, CMS has extensive forward calorimetry, which extends the coverage to $|\eta |<5.0$. Finally, muons are measured in gas-ionization detectors embedded in the steel flux-return yoke of the solenoid, with a coverage of $|\eta | < 2.4$. A two-tiered system, with a level-1 trigger followed by a high-level trigger (HLT), is used by CMS to record events of interest [41] for the offline analysis. A more detailed description of the CMS detector, together with a definition of the coordinate system used and the relevant kinematic variables, can be found in Ref. [42].

## Event selection and data unfolding

Events are reconstructed using a particle-flow algorithm [43] to identify and reconstruct individual particles from each collision by combining information from all CMS subdetectors. Identified particles include charged and neutral hadrons, electrons, muons, and photons. The particles are clustered into jets using the anti-$k_{\mathrm {T}}$ algorithm [44, 45] with a distance parameter of 0.4. In order to mitigate the effect of additional proton–proton interactions within the same or nearby bunch crossings (pileup) on the jet momentum measurement, the charged hadron subtraction technique [43] is used. Spurious jets from noise or non-collision backgrounds are rejected by applying jet identification criteria [46]. The jet energies are corrected for nonlinear and nonuniform response of the calorimeters through corrections obtained from data and Monte Carlo (MC) simulations [47]. To compare data with theoretical predictions, the same jet clustering algorithm is applied to the generated stable particles (lifetime $c\tau > 1\,\text {cm}$) from MC simulations with leading order (LO) pythia 8.212 [48, 49] predictions, and to the outgoing partons from next-to-leading (NLO) predictions.

The events used in this analysis are selected with triggers based upon either jet $p_{\mathrm {T}}$ or $H_{\mathrm {T}}$, as measured by the HLT, where $H_{\mathrm {T}}$ is the scalar sum of the $p_{\mathrm {T}}$ values of all the jets with $|\eta | < 3.0$ and $p_{\mathrm {T}}$ greater than 30GeV. The HLT selection requires having a jet with $p_{\mathrm {T}} >450\hbox {GeV}$ or an $H_{\mathrm {T}}$ value of at least 900GeV. The data sample was collected with the CMS detector in 2016 and corresponds to an integrated luminosity of 35.9$\,\text {fb}^{-1}$  [50].

In the subsequent offline analysis, events with a reconstructed primary vertex that lies within ± 24 $\text {\,cm}$ of the detector center along the beam line, and within 2$\text {\,cm}$ of the detector center in the plane transverse to the beam, are selected. The primary vertex is defined as the reconstructed vertex with the highest sum of the squares of all associated physics objects $p_{\mathrm {T}}$. The physics objects are the jets returned by the application of the anti-$k_{\mathrm {T}}$ algorithm to all tracks associated with the vertex, plus the corresponding associated missing transverse momentum, taken as the negative vector sum of the $p_{\mathrm {T}}$ of those jets.

The two leading jets are used to measure the dijet angular distributions in seven regions of the dijet invariant mass $M_{\mathrm {jj}}$. The $M_{\mathrm {jj}}$ regions, in units of TeV, are chosen to be 2.4–3.0, 3.0–3.6, 3.6–4.2, 4.2–4.8, 4.8–5.4, 5.4–6.0, and > 6.0. The highest $M_{\mathrm {jj}}$ range was chosen to maximize the expected sensitivity to the BSM signals considered. The phase space for this analysis is restricted by the requirements $\chi _{\text {dijet}} <16$ and $|y_{\text {boost}} |<1.11$, where $y_{\text {boost}} = (y_1 + y_2)/2$. This selection and the $M_{\mathrm {jj}}$ range definition restrict the absolute rapidities $|y_1 |$ and $|y_2 |$ of the two highest $p_{\mathrm {T}}$ jets to be less than 2.5 and their $p_{\mathrm {T}}$ to be larger than 200GeV. The trigger efficiency for events that satisfy the subsequent selection criteria exceeds 99% in all the $M_{\mathrm {jj}}$ ranges for the analysis. The observed numbers of events in the analysis phase space for each of the mass ranges are 353025, 71832, 16712, 4287, 1153, 330, and 95. The highest value of $M_{\mathrm {jj}}$ observed among these events is 8.2TeV.

In this paper, we present dijet angular distributions normalized to unity in each $M_{\mathrm {jj}}$ range, denoted $(1/\sigma _\text {dijet})(\mathrm {d}\sigma _\text {dijet}/\mathrm {d}\chi _\text {dijet})$, where $\sigma _\text {dijet}$ is the cross section in the analysis phase space.

Fluctuations in jet response from the resolution in jet $p_{\mathrm {T}}$ of the detector can cause lower energy jets to be misidentified as leading jets and also result in bin-to-bin event migrations in both $\chi _{\text {dijet}}$ and dijet mass. The corrections for these effects are obtained from a two-dimensional response matrix that maps the generator-level $M_{\mathrm {jj}}$ and $\chi _{\text {dijet}}$ distributions onto the measured values. This matrix is obtained using particle-level jets from the pythia MC event generator that are smeared in $p_{\mathrm {T}}$ using a double-sided Crystal Ball parameterization [51] of the response. This parameterization takes into account the full jet energy resolution, including non-Gaussian tails, and is derived from the full detector simulation. The width of the Gaussian core in the parameterization is adjusted to account for the difference in resolution observed between data and simulation [47]. The reason for deriving the response matrix from smeared generator-level MC rather than from full detector simulation is that significantly smaller statistical uncertainties can be achieved using the faster code. The measured distributions are unfolded to particle level by inverting the response matrix without regularization, using the RooUnfold package [52]. The unfolding changes the shape of the $\chi _{\text {dijet}}$ distributions by < 1% and < 8% across $\chi _{\text {dijet}}$ in the lowest and highest $M_{\mathrm {jj}}$ ranges, respectively. The fractions of event migrations between mass bins are 15–20% in the lowest $M_{\mathrm {jj}}$ range and 25–40% in the highest $M_{\mathrm {jj}}$ range, depending on $\chi _{\text {dijet}}$ values. The unfolding procedure was tested by splitting the simulation data into independent training and testing samples. The training sample was used to derive a response matrix and the smeared $\chi _{\text {dijet}}$ distributions from the test sample were unfolded using this response matrix. No significant difference was observed between the generated and unfolded $\chi _{\text {dijet}}$ distributions in the test sample. The effects of migrations between $\chi _{\text {dijet}}$ bins are negligible. The unfolding procedure is based on matrix inversion, while the procedure used in previous publications of dijet angular distributions [28, 29] was based on the D’Agostini iterative method [53]. We have compared these two methods by deriving limits from unfolded data, and the limits vary by less than 5%.

## Theoretical predictions

We compare the unfolded normalized dijet angular distributions with the predictions of perturbative QCD at NLO, available in nlojet++ 4.1.3 [54] in the fastnlo 2.1 framework [55]. EW corrections for dijet production [56] change the predicted normalized distributions by up to 1% (5%) for the lowest $\chi _{\text {dijet}}$ bins in small (large) values of $M_{\mathrm {jj}}$. The factorization ($\mu _{\mathrm {f}}$) and renormalization ($\mu _{\mathrm {r}}$) scales are set to the average $p_{\mathrm {T}}$ of the two jets, $\langle p_\mathrm {T} \rangle =(p_{\mathrm {T}} {}_{1}+p_{\mathrm {T}} {}_{2})/2$, and the PDFs are taken from the CT14 set [57]. The use of a more flexible statistical combination of multiple PDF sets as in PDF4LHC15_100 [57–62] exhibited small differences as compared to the CT14 PDF set. We evaluated the impact of nonperturbative effects from hadronization and multiple parton interactions on the QCD predictions using pythia with the CUETP8M1 tune [63] and herwig++ 2.7.1 [64] with tune EE5C [65]. The effects are found to be less than 1% and negligible for both MC generators.

The production and decay of the DM mediators in the simplified DM model are generated at LO using MadDM version 2.0.6 [66, 67] at fixed $g_{\mathrm {DM}}$ and $m_{\mathrm {DM}}$ values, where $g_{\mathrm {DM}} = 1.0$ and $m_{\mathrm {DM}} = 1\hbox {GeV}$. For these values of $g_{\mathrm {DM}}$ and $m_{\mathrm {DM}}$, the differences between vector and axial-vector mediators in the cross sections and in the acceptances are negligible in the analysis phase space.

BSM physics signatures from CIs with flavor-diagonal color-singlet couplings among quarks are described by the effective Lagrangian [7, 8]:

$$\begin{aligned} \begin{aligned} \mathcal {L}_{\mathrm {q}\mathrm {q}}&=\frac{2\pi }{\varLambda ^2} [ \eta _{\mathrm {LL}} ({\overline{\mathrm {q}}}_{\mathrm {L}}\gamma ^{\mu }\mathrm {q}_{\mathrm {L}})(\overline{\mathrm {q}} _{\mathrm {L}}\gamma _{\mu }\mathrm {q}_{\mathrm {L}}) \\&\quad +\,\eta _{\mathrm {RR}} (\overline{\mathrm {q}} _{\mathrm {R}}\gamma ^{\mu }\mathrm {q}_{\mathrm {R}})(\overline{\mathrm {q}} _{\mathrm {R}}\gamma _{\mu }\mathrm {q}_{\mathrm {R}}) \\&\quad +\,2\eta _{\mathrm {RL}} (\overline{\mathrm {q}} _{\mathrm {R}}\gamma ^{\mu }\mathrm {q}_{\mathrm {R}})(\overline{\mathrm {q}} _{\mathrm {L}}\gamma _{\mu }\mathrm {q}_{\mathrm {L}})], \end{aligned} \end{aligned}$$

where the subscripts $\mathrm {L}$ and $\mathrm {R}$ refer to the left and right chiral projections of the quark fields, respectively, and $\eta _{\mathrm {LL}}$, $\eta _{\mathrm {RR}}$, and $\eta _{\mathrm {RL}}$ are taken to be 0, ${+}\,1$, or ${-}\,1$ for the different combinations that correspond to different CI models. The following CI possibilities with color-singlet couplings among quarks are investigated:

**Table:** 

Model	$\left( \eta _{\mathrm {LL}},\,\eta _{\mathrm {RR}},\,\eta _{\mathrm {RL}} \right)$
$\varLambda ^{\pm }_{\mathrm {LL}}$	$(\pm \, 1, 0, 0)\,$
$\varLambda ^{\pm }_{\mathrm {RR}}$	$(0,\pm \, 1, 0)\,$
$\varLambda ^{\pm }_{\mathrm {VV}}$	$(\pm \, 1,\pm \, 1,\pm \, 1)\,$
$\varLambda ^{\pm }_{\mathrm {AA}}$	$(\pm \, 1,\pm \, 1,\mp \, 1)\,$
$\varLambda ^{\pm }_{(\mathrm {V-A})}$	$(0,0,\pm \, 1)\,$

The models with positive (negative) $\eta _{\mathrm {LL}}$ or $\eta _{\mathrm {RR}}$ lead to destructive (constructive) interference with the QCD terms, and consequently a lower (higher) cross section, respectively. In all CI models discussed in this paper, NLO QCD corrections are employed to calculate the cross sections. In proton–proton collisions, the $\varLambda ^{\pm }_{\mathrm {LL}}$ and $\varLambda ^{\pm }_{\mathrm {RR}}$ models result in identical lowest order cross sections and NLO corrections, and consequently lead to the same sensitivity. For $\varLambda ^{\pm }_{\mathrm {VV}}$ and $\varLambda ^{\pm }_{\mathrm {AA}}$, as well as for $\varLambda ^{\pm }_{(\mathrm {V-A})}$, the CI predictions are also identical at lowest order, but exhibit different NLO corrections and yield different sensitivities. The cijet 1.0 program [68] is used to calculate the CI terms, as well as the interference between the CI and QCD terms at NLO in QCD.

For the ADD model, two parameterizations for virtual graviton exchange are considered: Giudice–Rattazzi–Wells (GRW) [69] and Han–Lykken–Zhang (HLZ) [70]. In the GRW convention, the sum over the Kaluza–Klein graviton excitations in the effective field theory is regulated by a single cutoff parameter $\varLambda _{\mathrm {T}}$. In the HLZ convention, the effective theory is described in terms of two parameters, the cutoff scale $M_{\mathrm {S}}$ and the number of extra spatial dimensions $n_{\mathrm {ED}}$. The parameters $M_{\mathrm {S}}$ and $n_{\mathrm {ED}}$ are directly related to $\varLambda _{\mathrm {T}}$ [71]. We consider models with 2–6 EDs. The case of $n_{\mathrm {ED}} =1$ is not considered since it would require an ED of the size of the radius of the solar system; the gravitational potential at such distances would be noticeably modified, and this case is therefore excluded by observation. The case of $n_{\mathrm {ED}} =2$ is special in the sense that the relation between $M_{\mathrm {S}}$ and $\varLambda _{\mathrm {T}}$ also depends on the parton-parton CM energy $\sqrt{s}$. The ADD predictions are calculated using pythia.

Quantum black hole production is studied within the framework of the ADD model, with $n_{\mathrm {ED}} =6$ (ADD6), and the Randall–Sundrum model (RS1) [72, 73] with a single, warped extra dimension ($n_{\mathrm {ED}} =1$). In these models, the QBH production cross section depends on the mass of the QBH, ${M_\mathrm {Pl}}$, and the number of spatial dimensions. Since QBHs are produced with a mass threshold close to ${M_\mathrm {Pl}}$, we set the minimum QBH mass $M_{\mathrm {QBH}}$ equal to ${M_\mathrm {Pl}}$ for simplicity. The qbh 3.0 generator [74] is used for the predictions.

To take into account the NLO QCD and EW corrections to SM dijet production when probing the ADD, QBH, and DM models, the cross section difference $\sigma ^{\mathrm {QCD}}_{\text {NLO+EW corr}} - \sigma ^{\mathrm {QCD}}_{\text {LO}}$ is evaluated for each $M_{\mathrm {jj}}$ and $\chi _{\text {dijet}}$ bin and added to the SM+BSM predictions. This procedure provides an SM+BSM prediction where the QCD terms are corrected to NLO with EW corrections while the BSM terms are calculated at LO. While the ADD BSM prediction from pythia includes the interference terms of graviton exchange with QCD (obtained by subtracting the predictions $\sigma ^{\text {ADD+QCD}}_{\text {LO}} - \sigma ^{\text {QCD}}_{\text {LO}}$), the QBH and DM BSM predictions do not include such interference terms.

Exclusion limits on the BSM models studied in this paper are set based on the comparison of data that have not been corrected for resolution effects with both SM+BSM and SM predictions that have been folded to detector level. The comparison at detector level is done to eliminate some systematic uncertainties that are introduced during the unfolding procedure and simplifies the statistical evaluation. This procedure uses the same two-dimensional response matrix whose inverse is used for unfolding the data. It has been verified that the $\chi _{\text {dijet}}$ distributions for SM+BSM predictions folded with the response matrix derived from SM QCD multijet predictions smeared with the double-sided Crystal Ball parameterization of the jet $p_{\mathrm {T}}$ resolution agree with SM+BSM predictions smeared with this same parameterization. The folding procedure is equivalent to running the full detector simulation on the particle-level predictions, with the residual differences accounted for in the systematic uncertainties.

## Systematic uncertainties

The normalized $\chi _{\text {dijet}}$ distributions are relatively insensitive to many potential systematic effects. To present the uncertainties for the normalized shapes, the quoted values are reported for the lowest $\chi _{\text {dijet}}$ bins, where the uncertainties and potential contributions from BSM processes are typically the largest. The main experimental uncertainty is from the jet energy scale (JES) and the main theoretical uncertainty is from the choices of $\mu _{\mathrm {r}}$ and $\mu _{\mathrm {f}}$ scales.

### Experimental uncertainties

The overall JES uncertainty is less than 1%, and the variation of the JES as a function of pseudorapidity is less than 1% per unit $\eta$ [47, 75] in the phase space of the analysis. The JES uncertainties related to each step in the derivation of the $p_{\mathrm {T}}$ and $\eta$ dependent JES corrections are taken into account independently. In this way, the correlations of the JES uncertainty sources among the $M_{\mathrm {jj}}$ ranges and $\chi _{\text {dijet}}$ bins are included. For the purpose of display in figures and tables, the total JES uncertainty is obtained from the quadratic sum of these uncertainty sources and is found to be 3.6% in the lowest $M_{\mathrm {jj}}$ range and 9.2% in the highest $M_{\mathrm {jj}}$ range.

The uncertainty from the jet $p_{\mathrm {T}}$ resolution is evaluated by changing the width of the Gaussian core of the Crystal Ball parameterization of the response by up to ± 5% [47, 75], depending upon the jet $\eta$, and comparing the resultant distributions before and after these changes. This uncertainty is found to be less than 1% for all $M_{\mathrm {jj}}$. The uncertainty from the modeling of the tails of the jet $p_{\mathrm {T}}$ resolution [76] is evaluated using a Gaussian function to parameterize the response, and we assign an uncertainty equal to half of the difference between the distributions determined from this Gaussian ansatz and the nominal correction. The size of this uncertainty is less than 1.5% for all $M_{\mathrm {jj}}$.

Another source of uncertainty arises from the use of a parametric model to simulate the jet $p_{\mathrm {T}}$ resolution of the detector. This uncertainty is estimated by comparing the smeared $\chi _{\text {dijet}}$ distributions to the ones from a detailed simulation of the CMS detector using Geant4  [77], and is found to be 0.5% and 1% in the lowest and highest $M_{\mathrm {jj}}$ ranges, respectively.

In the unfolding procedure, there is an additional systematic uncertainty introduced due to potential mismodeling of the dijet kinematic distributions in pythia. This uncertainty is evaluated using MadGraph 5_amc@nlo 2.2.2 [78] predictions, as the kinematic distributions from MadGraph 5_amc@nlo and pythia are found to bracket the data. The inverted response matrix from pythia is applied to the smeared $\chi _{\text {dijet}}$ distributions from MadGraph 5_amc@nlo and the results are compared to the corresponding generated $\chi _{\text {dijet}}$ distributions. The differences are observed to be less than 1.5% for all $M_{\mathrm {jj}}$.

The effect from pileup is studied by comparing the $\chi _{\text {dijet}}$ distributions with various numbers of pileup interactions in simulated events. The numbers are varied according to the uncertainty of the total inelastic cross section of $\mathrm {p}\mathrm {p}$ collisions [79]. The effect on the $\chi _{\text {dijet}}$ distributions is observed to be negligible.

### Theoretical uncertainties

The uncertainties due to the choices of $\mu _{\mathrm {f}}$ and $\mu _{\mathrm {r}}$ scales in the NLO QCD predictions are evaluated by following the proposal in Refs. [80, 81] and changing the default choice of scales in the following 6 combinations: $(\mu _{\mathrm {f}}/\langle p_\mathrm {T} \rangle$, $\mu _{\mathrm {r}}/\langle p_\mathrm {T} \rangle )$ = (1 / 2, 1 / 2), (1 / 2, 1), (1, 1 / 2), (2, 2), (2, 1), and (1, 2). These changes modify the predictions of the normalized $\chi _{\text {dijet}}$ distributions by up to 8.5% and up to 19%, at small and large values of $M_{\mathrm {jj}}$, respectively. The uncertainty in the NLO QCD predictions due to the choice of PDFs is determined from the 28 eigenvectors of CT14 using the procedure described in Ref. [82], and is found to be less than 0.2% at low $M_{\mathrm {jj}}$ and less than 0.6% at high $M_{\mathrm {jj}}$. The uncertainty in the strong coupling constant has a negligible impact on the normalized $\chi _{\text {dijet}}$ distribution.

Scale and PDF uncertainties in the CI predictions are obtained using the same procedure as in the QCD predictions. In the ADD and QBH models, the scale and PDF uncertainties have a negligible impact on the limits as the signals only appear in the highest mass bins, where the statistical uncertainties dominate. The effect on the acceptance for the DM models due to the PDF uncertainty is evaluated using the 100 replica NNPDF3.0 PDF set [60] and found to be non-negligible in the $M_{\mathrm {jj}}$ ranges with $M_{\mathrm {jj}} >M_{\text {Med}}$ for DM mediators that have large mass and coupling. For example, for an axial-vector mediator with $M_{\text {Med}} = 6\hbox {TeV}$ and $g_{\mathrm {\mathrm {q}}} = 1.0$, which corresponds to a resonance with relative width of 50%, the uncertainty is 14% in the $M_{\mathrm {jj}} >\!6.0\hbox {TeV}$ bin.

Although the uncertainties are treated separately in the statistical analysis of the data, for display purposes in tables and figures we calculate the total experimental and theoretical uncertainty as the quadratic sum of the contributions due to the JES, the jet $p_{\mathrm {T}}$ resolution, the modeling of both the detector response and the dijet kinematics, and the contributions from $\mu _{\mathrm {f}}$, $\mu _{\mathrm {r}}$, and the PDFs. A summary of the leading experimental systematic uncertainties is provided in Table 1. The theoretical uncertainties quoted in the table apply to the QCD prediction. As shown in Table 1, systematic uncertainties dominate the total uncertainty in low $M_{\mathrm {jj}}$ regions, while the statistical uncertainty dominates in high $M_{\mathrm {jj}}$ regions.

**Table 1:** Summary of the leading experimental and theoretical uncertainties in the normalized $\chi _{\text {dijet}}$ distributions, in percent. While the statistical analysis represents each uncertainty through a change in the $\chi _{\text {dijet}}$ distribution correlated among all $\chi _{\text {dijet}}$ bins, this table summarizes each uncertainty by a representative value to show their relative contributions. For the lowest and highest dijet mass bins, the relative shift is given for the lowest $\chi _{\text {dijet}}$ bin. In the highest dijet mass bin, the dominant experimental contribution corresponds to the statistical uncertainty, while the dominant theoretical contribution is from the uncertainty in scales

Source of uncertainty	$2.4<M_{\mathrm {jj}} <3.0\hbox {TeV}$	$M_{\mathrm {jj}} >6.0\hbox {TeV}$
Statistical	0.7	27
JES	3.6	9.2
Jet $p_{\mathrm {T}}$ resolution (core)	1.0	1.0
Jet $p_{\mathrm {T}}$ resolution (tails)	1.0	1.5
Detector response model	0.5	1.0
Unfolding, model dependence	0.2	1.5
Total experimental	4.1	29
QCD NLO scale (6 changes in $\mu _{\mathrm {r}}$ and $\mu _{\mathrm {f}}$)	$_{-3.0}^{+8.5}$	$_{-5.8}^{+19}$
PDF (CT14 eigenvectors)	0.2	0.6
Total theoretical	8.5	19

## Results

**Fig. 1:**
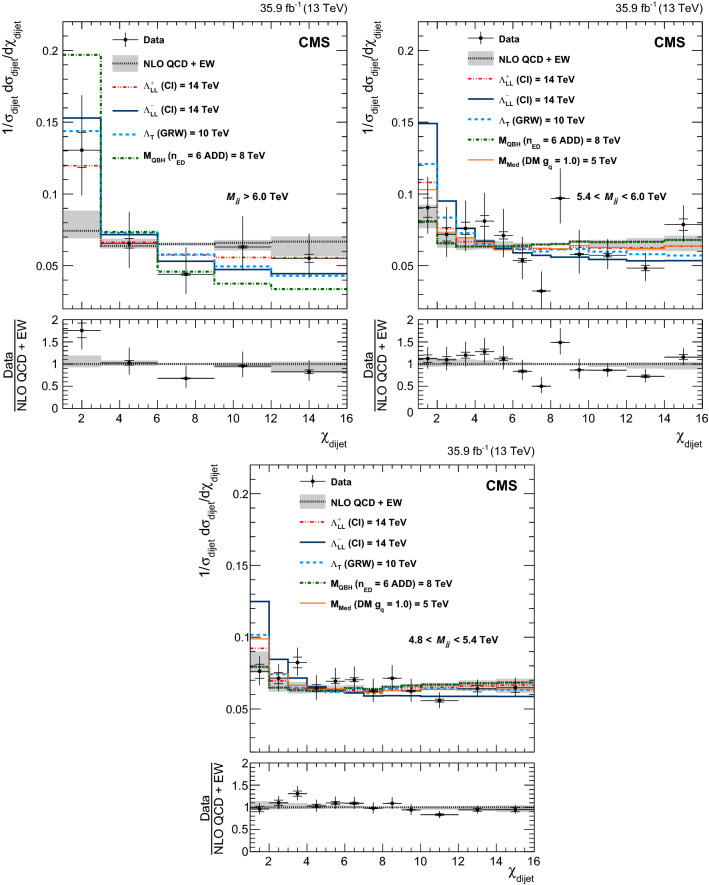
Normalized $\chi _{\text {dijet}}$ distributions in the three highest mass bins. Unfolded data are compared to NLO predictions (black dotted line). The error bars represent statistical and experimental systematic uncertainties combined in quadrature. The ticks on the error bars correspond to the experimental systematic uncertainties only. Theoretical uncertainties are indicated as a gray band. Also shown are the predictions for various CI, ADD, QBH, and DM scenarios. The lower panels show the ratio of the unfolded data distributions and NLO predictions

**Fig. 2:**
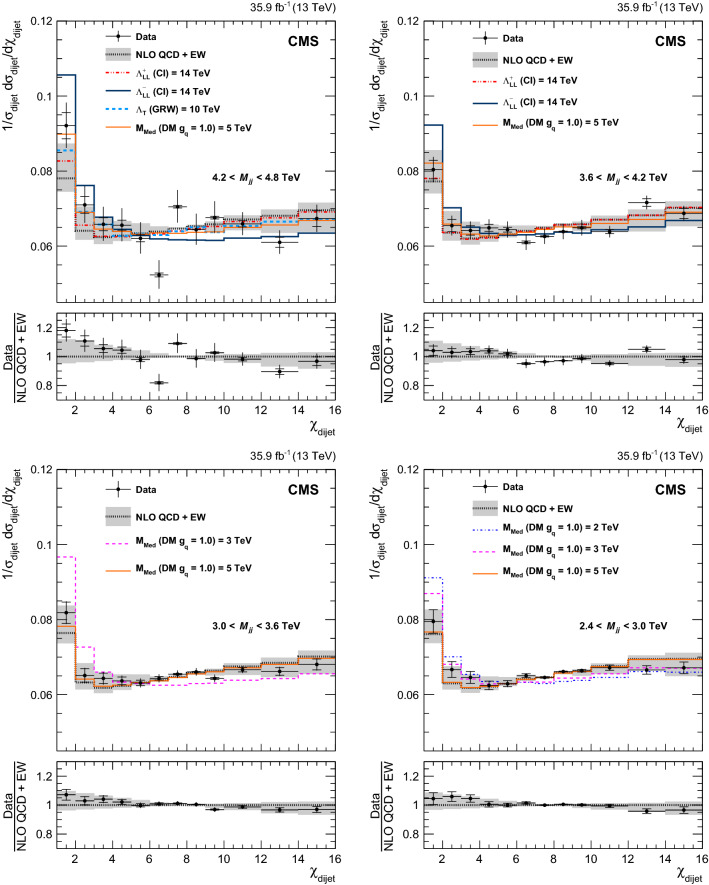
Normalized $\chi _{\text {dijet}}$ distributions in the four lower mass bins. Unfolded data are compared to NLO predictions (black dotted line). The error bars represent statistical and experimental systematic uncertainties combined in quadrature. The ticks on the error bars correspond to the experimental systematic uncertainties only. Theoretical uncertainties are indicated as a gray band. Also shown are the predictions for various CI, ADD, and DM scenarios. The lower panels show the ratio of the unfolded data distributions and NLO predictions

In Figs. 1 and 2 the measured normalized $\chi _{\text {dijet}}$ distributions for all mass bins unfolded to particle level are compared to NLO predictions with EW corrections. No significant deviation from the SM prediction is observed. The distributions are also compared to predictions for QCD+CI with CI scales equal to 14TeV, QCD+ADD with $\varLambda _{\mathrm {T}}\ (\mathrm {GRW})\ =10\hbox {TeV}$, QCD+QBH with $M_{\mathrm {QBH}} ~(\mathrm {ADD6}) = 8\hbox {TeV}$, and QCD+DM with $M_{\mathrm {Med}}=2$, 3 and 5TeVand $g_{\mathrm {\mathrm {q}}} =1.0$. The signal distributions are only shown for the $M_{\mathrm {jj}}$ ranges that contribute to the sensitivity for the BSM searches.

The asymptotic approximation [83] of the $\mathrm {\text {CL} _s}$ criterion [84, 85] is used to set exclusion limits on the parameters for the BSM models [86]. The limits obtained using this approximation were tested against the $\mathrm {\text {CL} _s}$ limits obtained using ensembles of pseudo experiments for several of the models examined, and the differences were found to be negligible. The likelihoods $L_\mathrm {QCD}$ and $L_\mathrm {QCD+BSM}$ are defined for the respective QCD-only and QCD+BSM hypotheses as a product of Poisson likelihood functions for each bin in $\chi _{\text {dijet}}$. The predictions for each $M_{\mathrm {jj}}$ range are normalized to the number of observed events in that range. Systematic uncertainties are treated as nuisance parameters in the likelihood model. Following Ref. [17], the nuisance parameters are profiled with respect to the QCD-only and QCD+BSM models by maximizing the corresponding likelihood functions. The *p*-values for the two hypotheses, $P_\mathrm {QCD+BSM}(q \ge q_\text {obs})$ and $P_\mathrm {QCD}(q \le q_\text {obs})$, are evaluated for the profile log-likelihood ratio $q = -\,2 \ln ({L_\text {QCD+BSM}}/{L_\mathrm {QCD}})$. Limits on the QCD+BSM models are set based on the quantity $\mathrm {\text {CL}}_\mathrm {s} = P_\mathrm {QCD+BSM}(q\ge q_\text {obs}) / (1-P_\mathrm {QCD}(q \le q_\text {obs}))$, which is required to be less than 0.05 for a 95% confidence level ($\text {CL}$) of exclusion. Because of the large number of events in the low-$M_{\mathrm {jj}}$ range, which constrain the systematic uncertainties, we obtain 2–30% better observed limits on the BSM scales and masses compared to the limits obtained using the method in the predecessor of this search reported in Ref. [29], where the nuisance parameters were marginalized rather than profiled.

In the limit calculations, not all $M_{\mathrm {jj}}$ ranges are included in the likelihoods; only those that improve the expected limits by more than 1% are used. We use mass bins with $M_{\mathrm {jj}} >3.6\hbox {TeV}$ for the CI models, $M_{\mathrm {jj}} >4.2\hbox {TeV}$ for the ADD models, and $M_{\mathrm {jj}} >4.8\hbox {TeV}$ for the QBH models. For the DM mediators, we use mass bins that cover the $M_{\mathrm {jj}}$ range of 0.5$M_{\text {Med}}$–1.2$M_{\text {Med}}$. The exclusion limits on the BSM models are determined using detector-level $\chi _{\text {dijet}}$ distributions and theoretical predictions at detector level. By using the detector-level $\chi _{\text {dijet}}$ distributions, each bin of the $\chi _{\text {dijet}}$ distributions can be modeled by a Poisson likelihood function, while at particle level, the unfolded data distributions have correlations among the dijet mass bins. As a cross-check, the limits are also determined for the case where the unfolded $\chi _{\text {dijet}}$ distributions, approximated by Poisson likelihood functions, and particle-level theoretical predictions are used in the limit extraction procedure. The resulting observed limits on the BSM scales and masses are found to be more stringent than those determined at detector level by 1–10%, depending on the model. The agreement of the data with QCD predictions is quantified by calculating $P_\text {QCD}(q < q_\text {obs})$ for each mass bin separately. The largest excess is found in the first data point of the > 6.0TeVmass bin, with a significance of 1.8 standard deviations. When combining mass bins in the various QCD+BSM models under study, the largest significances are found to be 2.7–2.8 standard deviations for the QCD+DM model with $M_{\text {Med}} = 4.5$–6TeVand $g_{\mathrm {\mathrm {q}}} =1.0$.

**Fig. 3:**
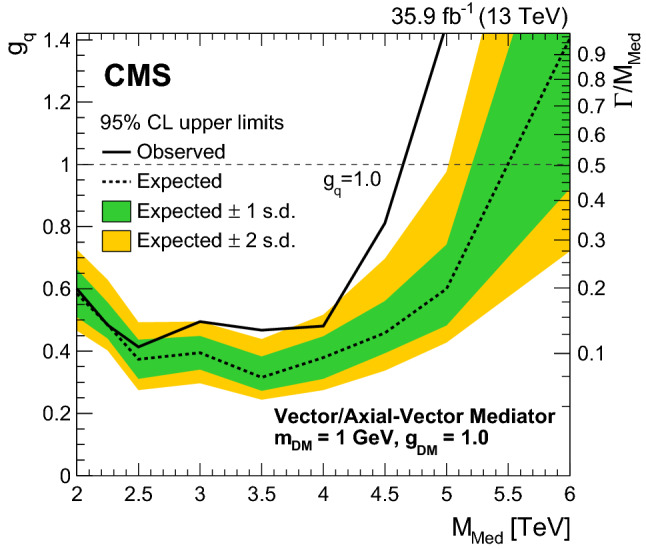
The 95% $\text {CL}$ upper limits on the quark coupling $g_{\mathrm {\mathrm {q}}}$, as a function of mass, for an axial-vector or vector DM mediator with $g_{\mathrm {DM}} =1.0$ and $m_{\mathrm {DM}} =1\hbox {GeV}$. The observed limits (solid), expected limits (dashed) and the variation in the expected limit at the 1 and 2 standard deviation levels (shaded bands) are shown. A dotted horizontal line shows the coupling strength for a benchmark DM mediator with $g_{\mathrm {\mathrm {q}}} =1.0$. The corresponding limits on the width of the mediators are shown on the vertical axis on the right-hand side of the figure

Figure 3 shows the 95% $\text {CL}$ upper limits on $g_{\mathrm {\mathrm {q}}}$ as a function of the mass of the vector or axial-vector DM mediator with $g_{\mathrm {DM}} =1.0$ and $m_{\mathrm {DM}} =1\hbox {GeV}$. The corresponding limits on the width of the mediators are shown on the vertical axis on the right-hand side of Fig. 3. The degradation of the limits below $M_{\text {Med}} =2.5\hbox {TeV}$ and above $M_{\text {Med}} =4\hbox {TeV}$ can be explained as follows. For resonance masses below the lower $M_{\mathrm {jj}}$ boundary of the analysis at 2.4TeV, the acceptance increases rapidly as a function of resonance mass (e.g., from 1.4% at $M_{\text {Med}} =2\hbox {TeV}$ to 16% at $M_{\text {Med}} =2.5\hbox {TeV}$, for $g_{\mathrm {\mathrm {q}}} =0.5$), resulting in the improvement of the limit on $g_{\mathrm {\mathrm {q}}}$ as a function of resonance mass. For large values of resonance mass and width (e.g., for $M_{\text {Med}} >4\hbox {TeV}$ and $g_{\mathrm {\mathrm {q}}} >0.5$), the mediator is primarily produced off-shell with a mass less than the $M_{\mathrm {jj}}$ boundary of the analysis at 2.4TeV. The acceptance for high resonance masses thus decreases as a function of resonance width (e.g., for $M_{\text {Med}} =5\hbox {TeV}$, from 25% at $g_{\mathrm {\mathrm {q}}} =0.5$ to 8% at $g_{\mathrm {\mathrm {q}}} =1.5$), resulting in the fast deterioration of the limit on $g_{\mathrm {\mathrm {q}}}$ at high resonance masses. The observed limit above 5TeVis at $\varGamma /M_{\mathrm {Med}} \ge 1$, thus in a region where the simplified model of a mediator particle is no longer valid. For $M_{\text {Med}}$ between 2.0 and 4.6TeV, this search excludes couplings $1.0 \le g_{\mathrm {\mathrm {q}}} \le 1.4$, which are not accessible via dijet resonance searches.

**Fig. 4:**
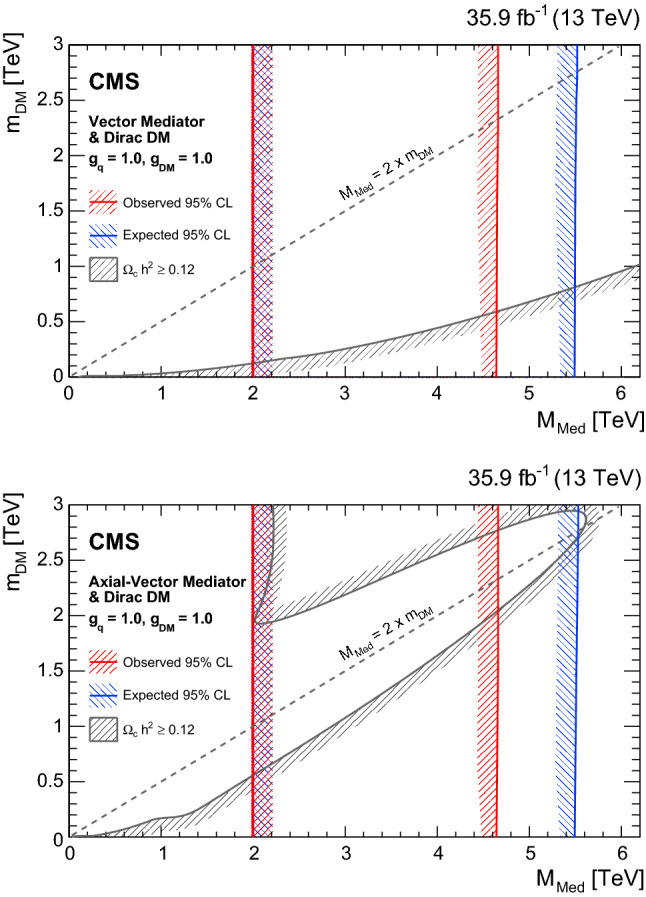
The 95% $\text {CL}$ observed (red) and expected (blue) excluded regions in the plane of $m_{\mathrm {DM}}$ and $M_{\text {Med}}$, for a vector mediator (upper) and an axial-vector mediator (lower) for a DM benchmark model with $g_{\mathrm {DM}} =g_{\mathrm {\mathrm {q}}} =1.0$. These are compared to constraints from the cosmological relic density of DM (gray) determined from astrophysical measurements [87], using MadDM. In the hatched area, DM is overabundant. The observed and expected lower bounds for $M_{\text {Med}}$ overlap with each other

The limits for $M_{\text {Med}}$ at arbitrary $m_{\mathrm {DM}}$ and $g_{\mathrm {DM}}$ can be calculated based on the fact that at fixed mediator production cross sections, changes in the width of the DM decay channel will lead to changes in the width of the quark decay channel. For the models with $g_{\mathrm {\mathrm {q}}} =1.0$, $g_{\mathrm {DM}} =1.0$, and $2m_{\mathrm {DM}} <M_{\text {Med}}$, in which the total width of the mediator is dominated by the width of the quark decay channel due to the large number of possible quark flavors and colors, the exclusion range for $M_{\text {Med}}$ has little dependence on $m_{\mathrm {DM}}$. For the models with $2m_{\mathrm {DM}} \ge M_{\text {Med}}$, the width of the DM decay channel is assumed to be zero. The resulting exclusion regions for vector and axial-vector mediators with $g_{\mathrm {\mathrm {q}}} =1.0$ and $g_{\mathrm {DM}} =1.0$ in the $m_{\mathrm {DM}}$ and $M_{\text {Med}}$ plane are shown in Fig. 4.

The observed and expected exclusion limits at 95% $\text {CL}$ on different CI, ED, QBH, and DM models obtained in this analysis are listed in Table 2. The observed limits are less stringent than the expected limits because of the upward fluctuation in the measured distributions compared to the theoretical predictions. The limits on all models are more stringent than those obtained from data collected by CMS in 2015 [29].

**Table 2:** Observed and expected exclusion limits at 95% $\text {CL}$ for various CI, ADD, QBH, and DM models. The 68% ranges of expectation for the expected limit are given as well. For the DM vector mediator, couplings $g_{\mathrm {DM}} =1.0$, $g_{\mathrm {\mathrm {q}}} \ge 1$ and a DM mass of 1GeVare assumed and a range of masses instead of a lower limit is quoted

Model	Observed lower limit (TeV)	Expected lower limit (TeV)
CI		
$\varLambda _{\mathrm {LL/RR}}^{+}$	12.8	$14.6 \pm 0.8$
$\varLambda _{\mathrm {LL/RR}}^{-}$	17.5	$23.5 \pm 3.0$
$\varLambda _{\mathrm {VV}}^{+}$	14.6	$16.4 \pm 0.8$
$\varLambda _{\mathrm {VV}}^{-}$	22.4	$30.7 \pm 3.7$
$\varLambda _{\mathrm {AA}}^{+}$	14.7	$16.5 \pm 0.8$
$\varLambda _{\mathrm {AA}}^{-}$	22.3	$30.6 \pm 3.8$
$\varLambda _{\mathrm {(V-A)}}^{+}$	9.2	$11.5 \pm 1.0$
$\varLambda _{\mathrm {(V-A)}}^{-}$	9.3	$11.8 \pm 1.1$
ADD		
$\varLambda _\mathrm {T}$ (GRW)	10.1	$11.4 \pm 0.9$
$M_{\mathrm {S}}$ (HLZ) $n_{\mathrm {ED}} =2$	10.7	$12.4 \pm 1.0$
$M_{\mathrm {S}}$ (HLZ) $n_{\mathrm {ED}} =3$	12.0	$13.6 \pm 1.1$
$M_{\mathrm {S}}$ (HLZ) $n_{\mathrm {ED}} =4$	10.1	$11.4 \pm 0.9$
$M_{\mathrm {S}}$ (HLZ) $n_{\mathrm {ED}} =5$	9.1	$10.3 \pm 0.8$
$M_{\mathrm {S}}$ (HLZ) $n_{\mathrm {ED}} =6$	8.5	$9.6 \pm 0.8$
QBH		
$M_{\mathrm {QBH}}$ (ADD $n_{\mathrm {ED}} =6$)	8.2	$8.5 \pm 0.4$
$M_{\mathrm {QBH}}$ (RS $n_{\mathrm {ED}} =1$)	5.9	$6.3 \pm 0.7$
DM		
Vector/axial-vector $M_{\text {Med}}$	2.0–4.6	2.0–5.5

## Summary

A search has been presented for physics beyond the standard model, based on normalized dijet angular distributions obtained in 2016 from proton–proton collisions at the LHC. The data sample corresponds to an integrated luminosity of 35.9$\,\text {fb}^{-1}$. The angular distributions, measured over a wide range of dijet invariant masses, are found to be in agreement with the predictions of perturbative quantum chromodynamics. The results are used to set 95% confidence level lower limits on the contact interaction scale for a variety of quark compositeness models, the ultraviolet cutoff in models of extra spatial dimensions, the minimum mass of quantum black holes, and the mass and couplings in dark matter models. For the first time, lower limits between 2.0 and 4.6TeVare set on the mass of a dark matter mediator for (axial-)vector mediators, for the universal quark coupling $1.0\le g_{\mathrm {\mathrm {q}}} \le 1.4$. This region is not accessible through dijet resonance searches. The lower limits for the contact interaction scale $\varLambda$ range from 9.2 to 22.4TeV. The lower limits on the ultraviolet cutoff in the Arkani–Hamed–Dimopoulos–Dvali model are in the range of 8.5–12TeV, and are the most stringent limits available. Quantum black hole masses below 8.2TeVare excluded in the model with six large extra spatial dimensions, and below 5.9TeVin the Randall–Sundrum model with a single, warped extra dimension. To facilitate comparisons with the predictions of other models, the angular distributions, corrected to particle level, are published in HEPData.
